# Supplementary material for: Global, regional, and national estimates of the impact of a maternal Klebsiella pneumoniae vaccine: A Bayesian modeling analysis
Source: PLoS Med. 2023 May 22;20(5):e1004239. doi: 10.1371/journal.pmed.1004239 (PMC10270628; doi:10.1371/journal.pmed.1004239)
Supplement: S1 Text — Fig A. Raw data. Calculated percent of neonatal sepsis deaths that are associated (i.e., an isolate from the neonate who died was culture-positive) with various etiologies across each study by location. Table A. Number of neonates who died of neonatal sepsis divided by number of neonates surveilled by location. Fig B. Flow diagram summarizing cases of culture-confirmed sepsis used in the main analysis of vaccine-avertable sepsis and AMR. Fig C. Flow diagram summarizing data collection and cleaning of the Klebsiella pneumoniae genomes used in the antimicrobial resistance genes (ARG) prevalence analysis. BARNARDS refers to data gathered from the Burden of Antimicrobial Resistance in Neonates in Developing Societies study. Fig D. Distribution of available genomic data from PathogenWatch and the Burden of Antimicrobial Resistance in Neonates from Developing Societies study by year used in the antimicrobial resistance gene prevalence analysis. Fig E. Tree map of the distribution of available K. pneumoniae isolates across countries for use in the prevalence of antimicrobial resistance genes analysis. Colors have no meaning and are used to create contrast between countries. Fig F. Schematic diagram of the modeling framework. Table B. Model parameters. Note that this refers to values that are used as inputs to various modeling stages, not quantities that are predicted through the modeling process described in Fig F. Fig G. Raw resistance data by study. Table C. Regression analysis results for the model used to extrapolate the number of averted deaths from those countries for which we have data to all countries. Fig H. 2.5th percentile (i.e., lower bound) of estimates represented as maps shown in Fig 3. The maps are reprinted from pygal_maps_world under GNU GPL. Fig I. 97.5th percentile (i.e., lower bound) of estimates represented as maps shown in Fig 3. The maps are reprinted from pygal_maps_world under GNU GPL. Fig J. As Fig 3C and 3D but for ampicillin. Median estimates s [file pmed.1004239.s001.pdf]

Global, regional, and national estimates of the impact of a maternal *Klebsiella pneumoniae* vaccine: A Bayesian modeling analysis

Supplementary Materials

Chirag K. Kumar[1], Kirsty Sands[2], Timothy R. Walsh[2,], Seamus O'Brien[3], Mike Sharland[4], Joseph A. Lewnard[5], Hao Hu[6], Padmini Srikantiah[6], Ramanan Laxminarayan[1, 7*]

1 Princeton University, Princeton, New Jersey, USA

2 Ineos Oxford Institute for Antimicrobial Resistance, Department of Zoology, Oxford, UK

3 Global Antibiotic Research and Development Partnership, Geneva, Switzerland

4 Center for Neonatal and Paediatric Infection (CNPI), Institute of Infection and Immunity, St George's University of London, London, UK

5 Division of Epidemiology, School of Public Health, University of California at Berkeley, Berkeley, California, USA

6 Bill and Melinda Gates Foundation, Seattle, Washington, USA

7 One Health Trust, Bengaluru, India

* ramanan@onehealthtrust.org

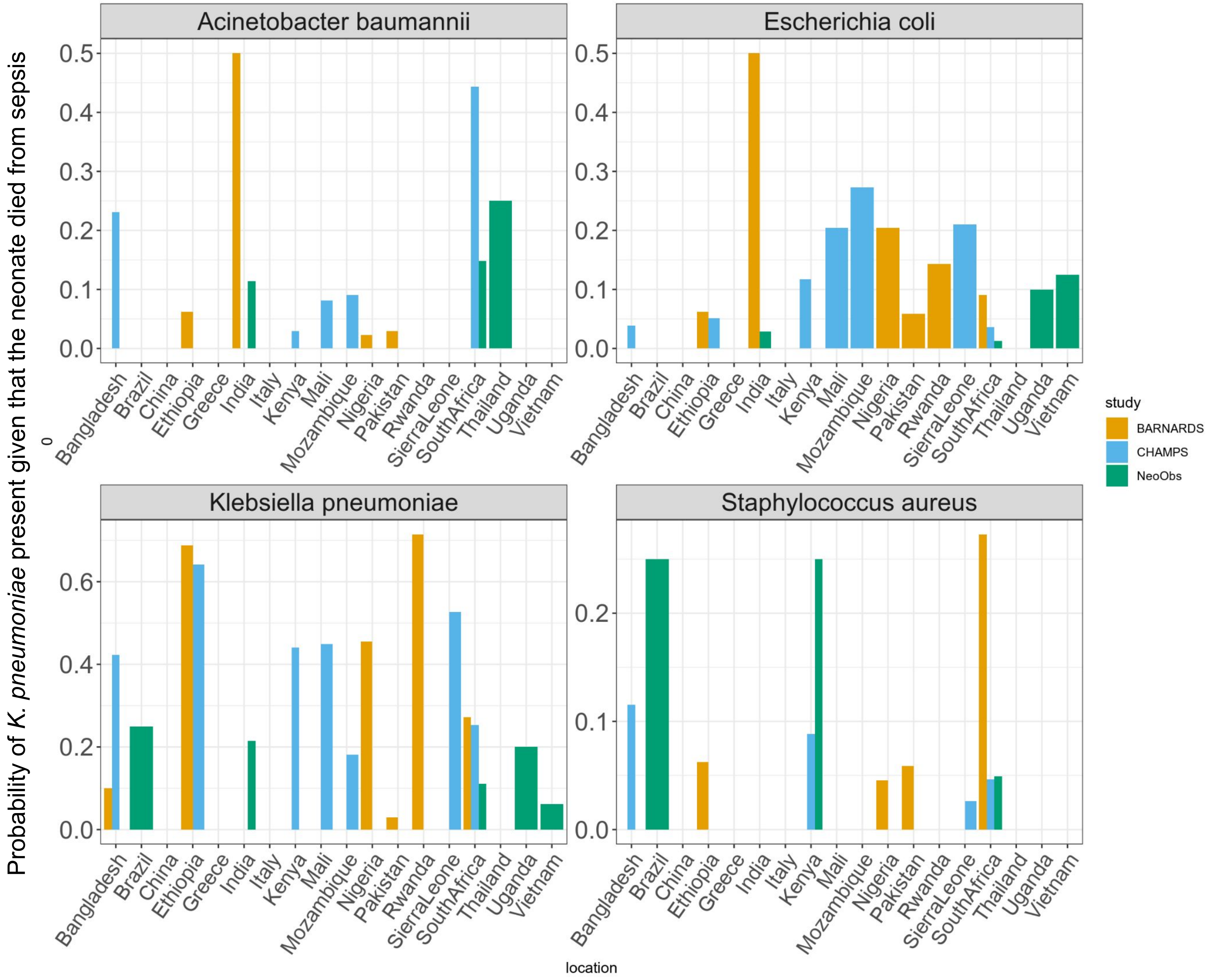

Figure A. Raw data of species identification on bacterial isolates. Calculated percent of neonatal sepsis deaths that are associated (i.e., an isolate from the neonate who died was culture-positive) with various etiologies across each study by location.

**Country**	CHAMPS	BARNARDS	NeoObs
Bangladesh	11/26	1/10	0/2
Brazil	–	–	1/4
China	–	–	0/6
Ethiopia	25/39	11/16	–
Greece	–	–	0/2
India	–	0/2	15/70
Italy	–	–	0/0
Kenya	15/34	–	0/4
Mali	22/49	–	–
Mozambique	2/11	–	–
Nigeria	–	20/44	–
Pakistan	–	1/34	–
Rwanda	–	5/7	–
Sierra Leone	20/38	–	–
South Africa	104/410	3/11	9/81
Thailand	–	–	0/4
Uganda	–	–	2/10
Vietnam	–	–	1/16

Table A. Number of neonates who died of neonatal sepsis divided by number of neonates surveilled by location.

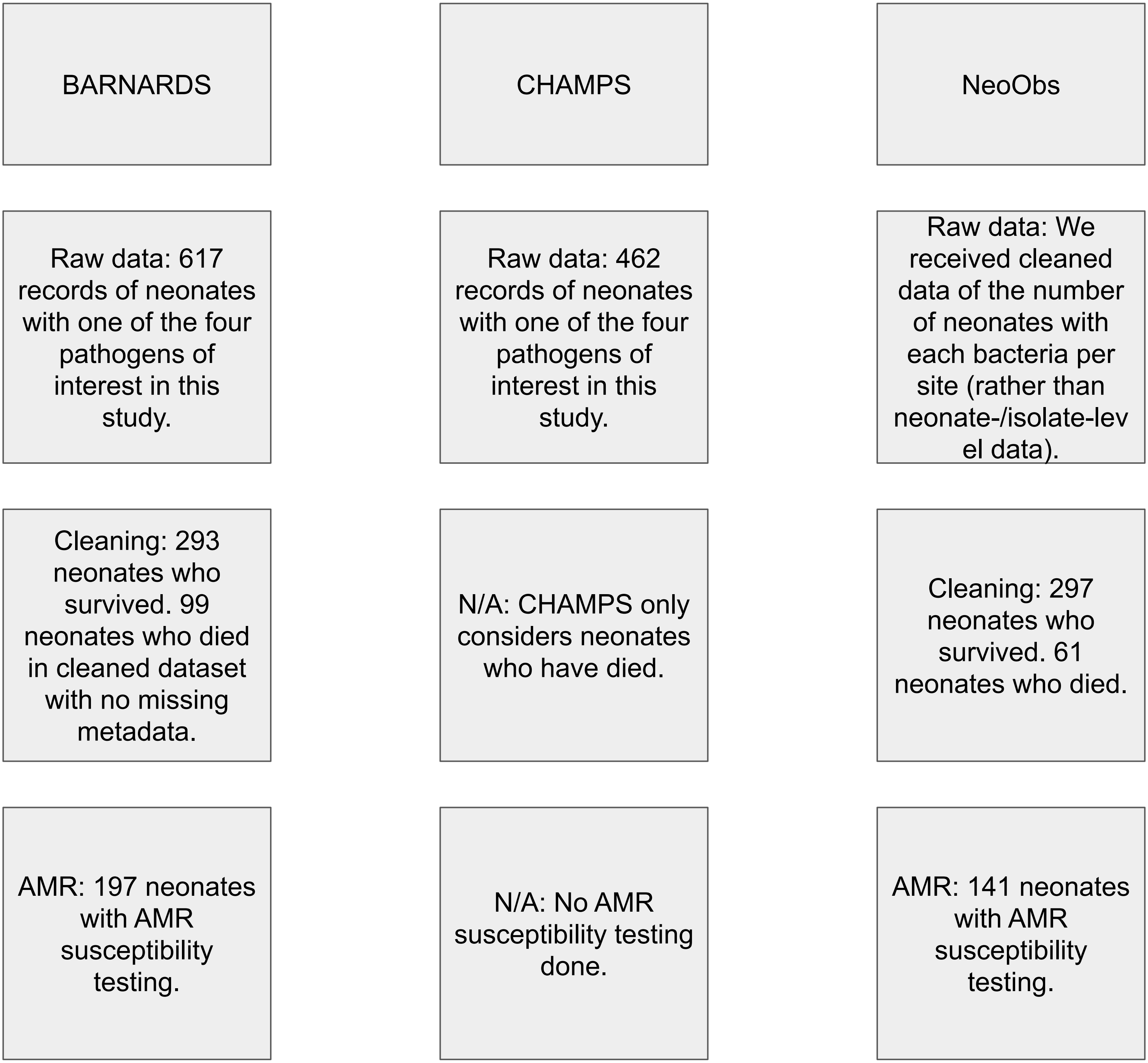

Figure B. Flow diagram summarizing cases of culture-confirmed sepsis used in the main analysis of vaccine-avertable sepsis and AMR.

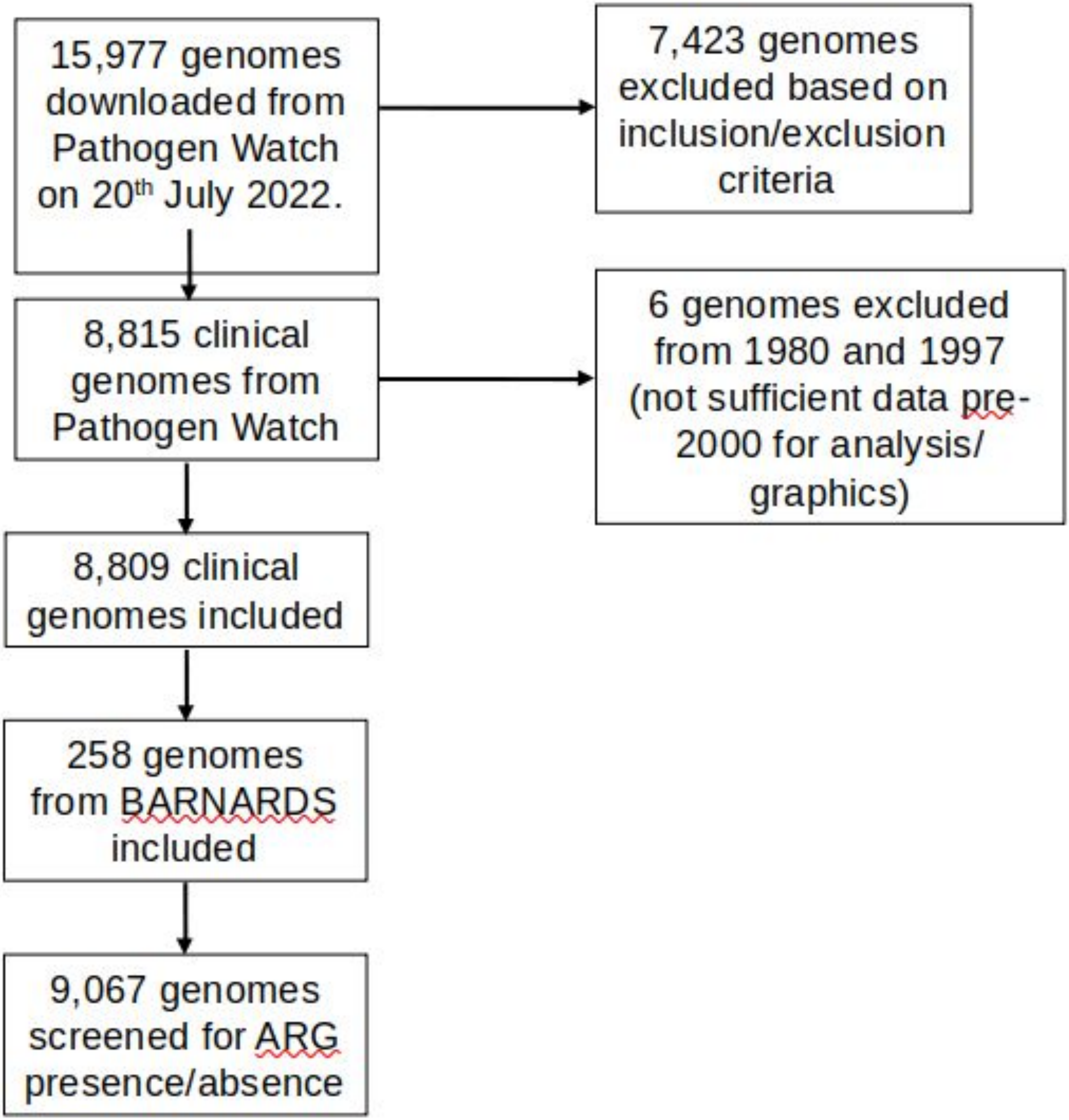

Figure C. Flow diagram summarizing data collection and cleaning of the *Klebsiella pneumoniae* genomes used in the antimicrobial resistance genes (ARG) prevalence analysis. BARNARDS refers to data gathered from the Burden of Antimicrobial Resistance in Neonates in Developing Societies study.

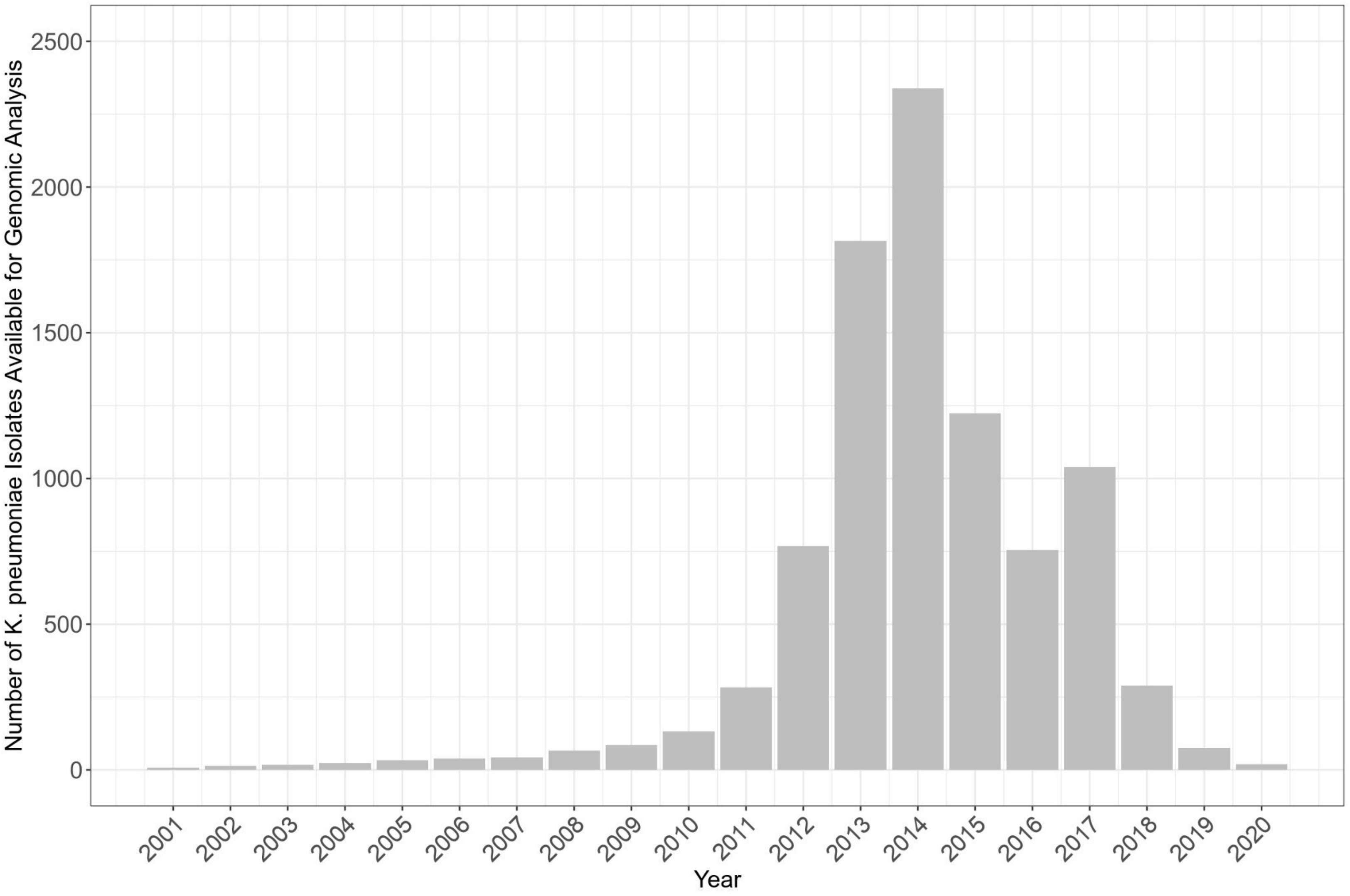

Figure D. Distribution of available genomic data from PathogenWatch and the Burden of Antimicrobial Resistance in Neonates from Developing Societies study by year used in the antimicrobial resistance gene prevalence analysis.

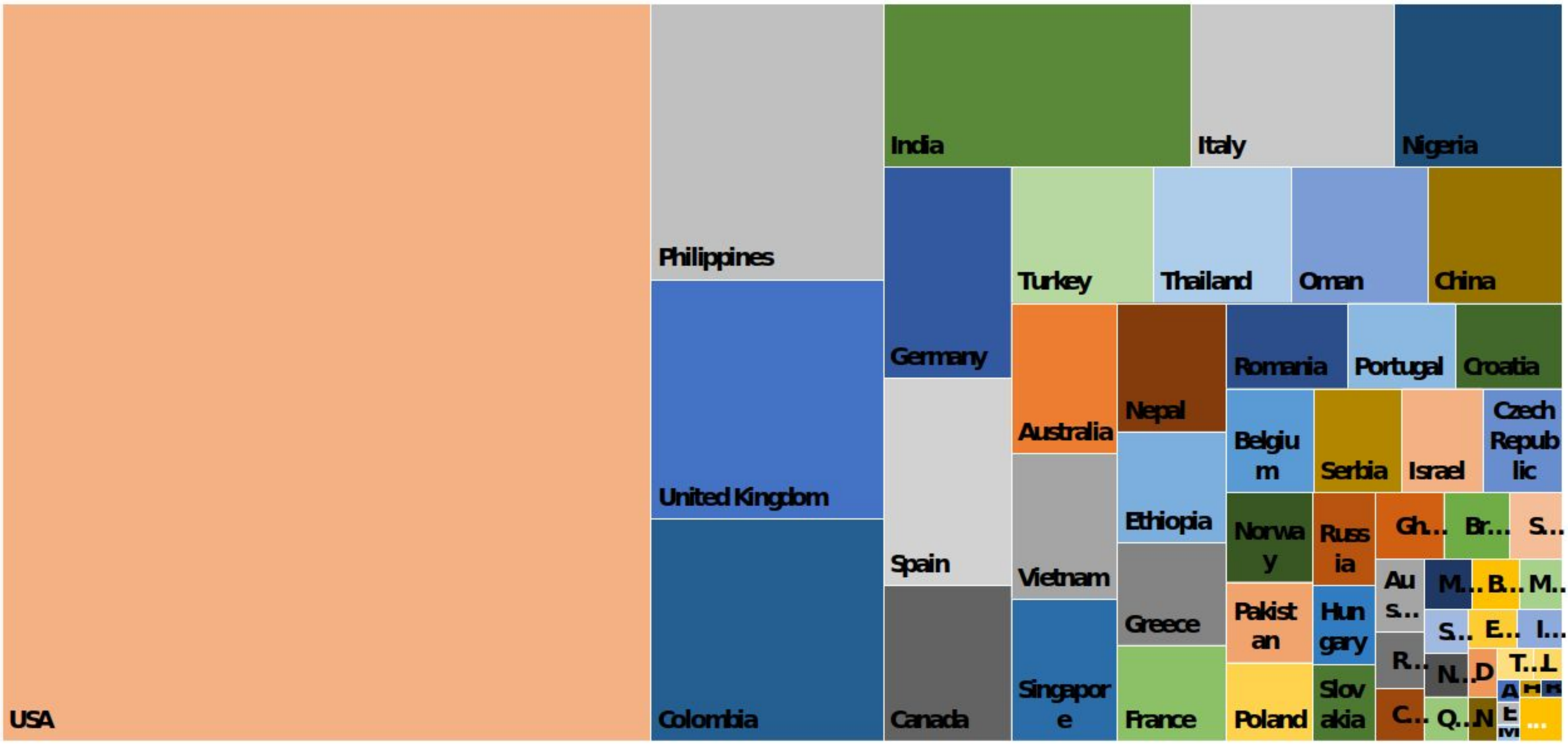

Figure E. Tree map of the distribution of available *K. pneumoniae* isolates across countries for use in the prevalence of antimicrobial resistance genes analysis. Colors are have no meaning and are used to create contrast between countries.

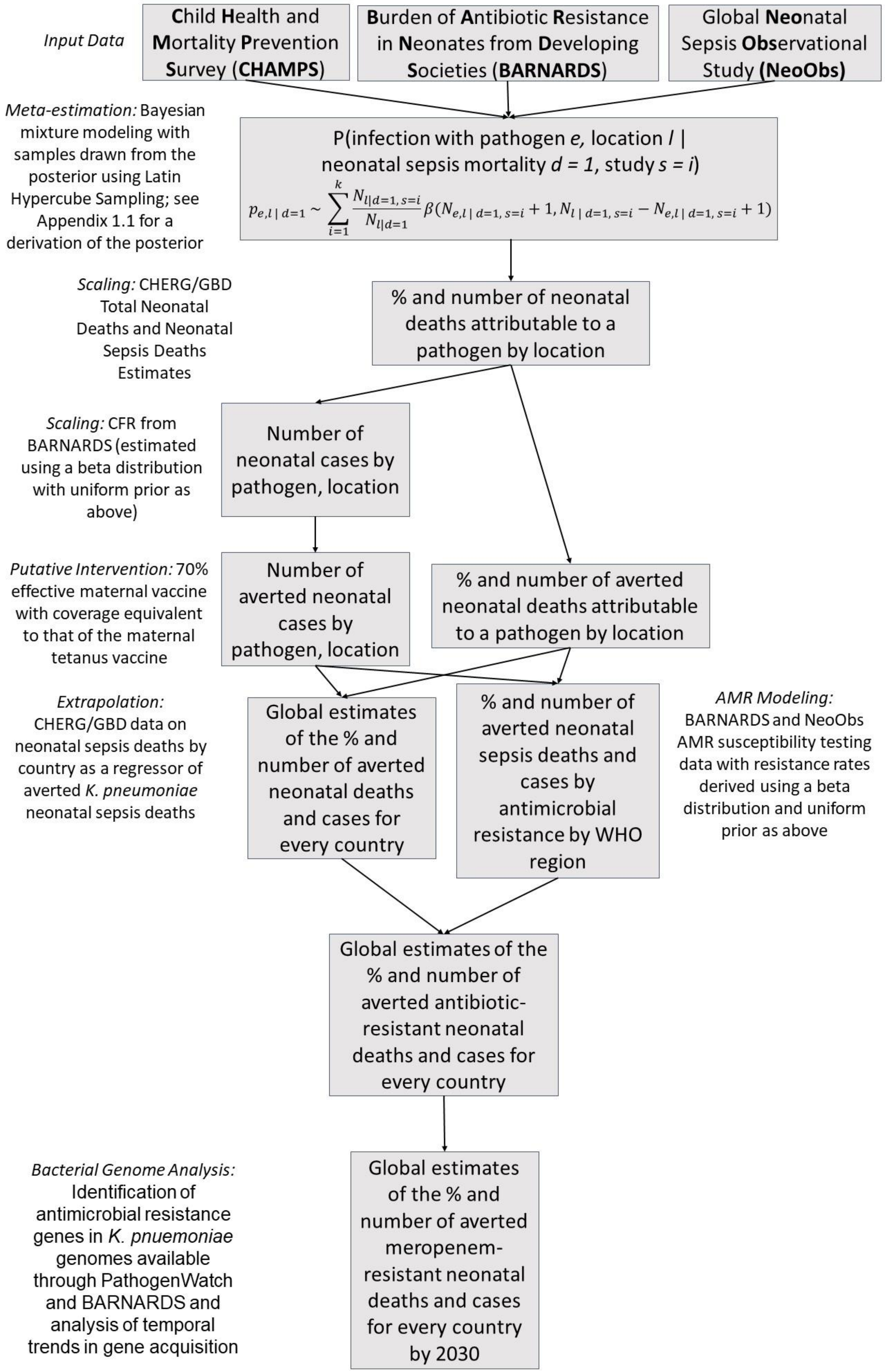

Figure F. Schematic diagram of the modeling framework.

**Parameter**	Value	Reference	Notes
Number of neonatal sepsis deaths and total neonatal deaths	Varies by location	CHERG (Child Health and Epidemiology Reference Group) and GBD (Global Burden of Disease)	
Vaccine efficacy	70%		Assumed
Vaccine coverage	Range: 38.5% to 100%; Median: 90%		Equivalent to that of maternal tetanus vaccine
Average yearly rate of increase in unique carbapenem antimicrobial resistance genes	0.0497 [0.042, 0.058]		Derived through regression equations between number of unique carbapenem resistance genes per bacteria and year

Table B. Model parameters. Note that this refers to values that are used as inputs to various modeling stages, not quantities that are predicted through the modeling process described in Fig. F.

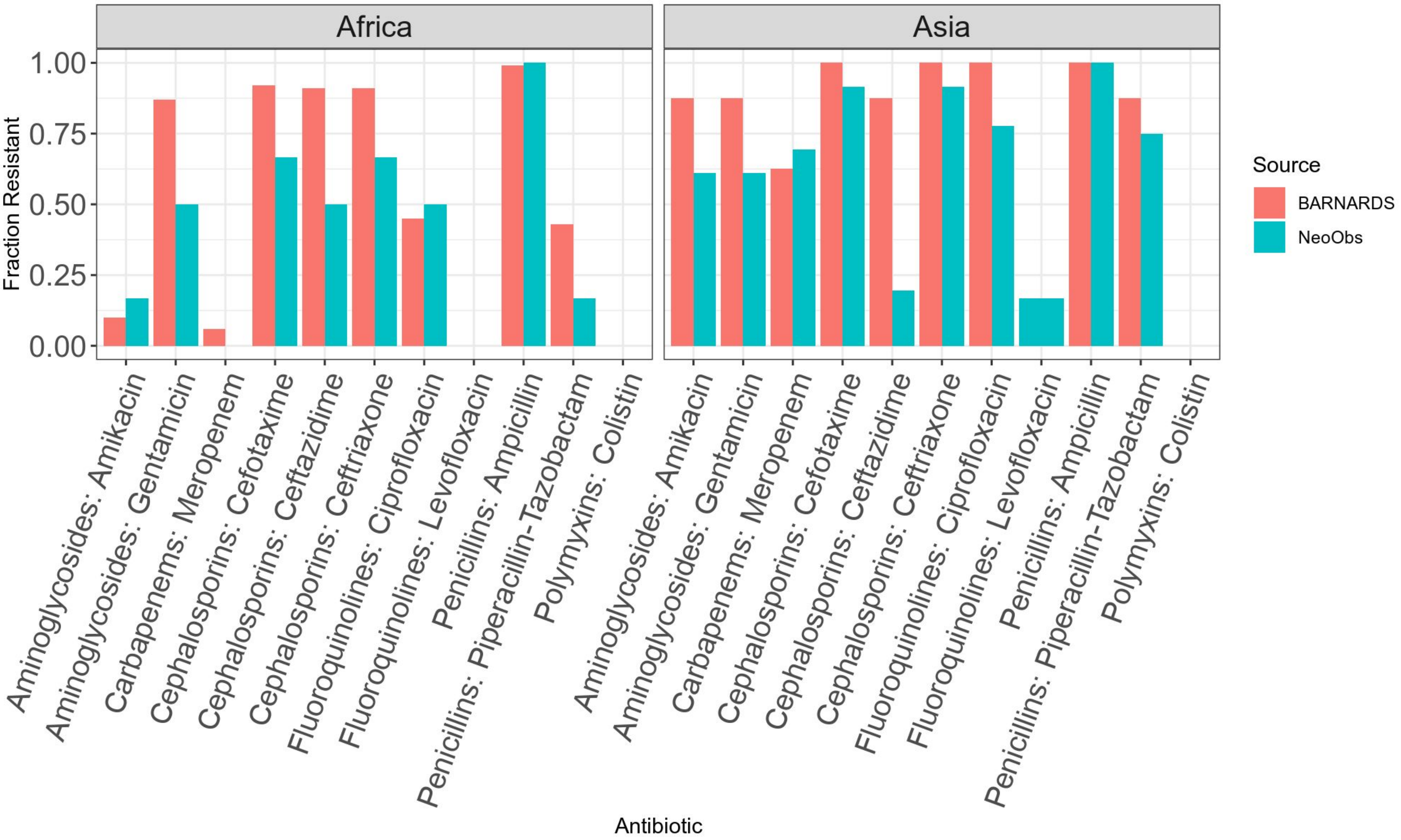

Figure G. Raw resistance data by study.

Predictand: Number of averted neonatal sepsis deaths	Independent variable: Number of neonatal sepsis deaths from CHERG	Model: Ordinary Least Squares
R^2: 0.840	Coefficient: 0.1405<br>Standard error: 0.016<br>95%tile Confidence interval: 0.107 - 0.174	
F-stat: 78.84; p-value: 233e07	t-val: 8.879	

Table C. Regression analysis results for the model used to extrapolate the number of averted deaths from those countries for which we have data to all countries.

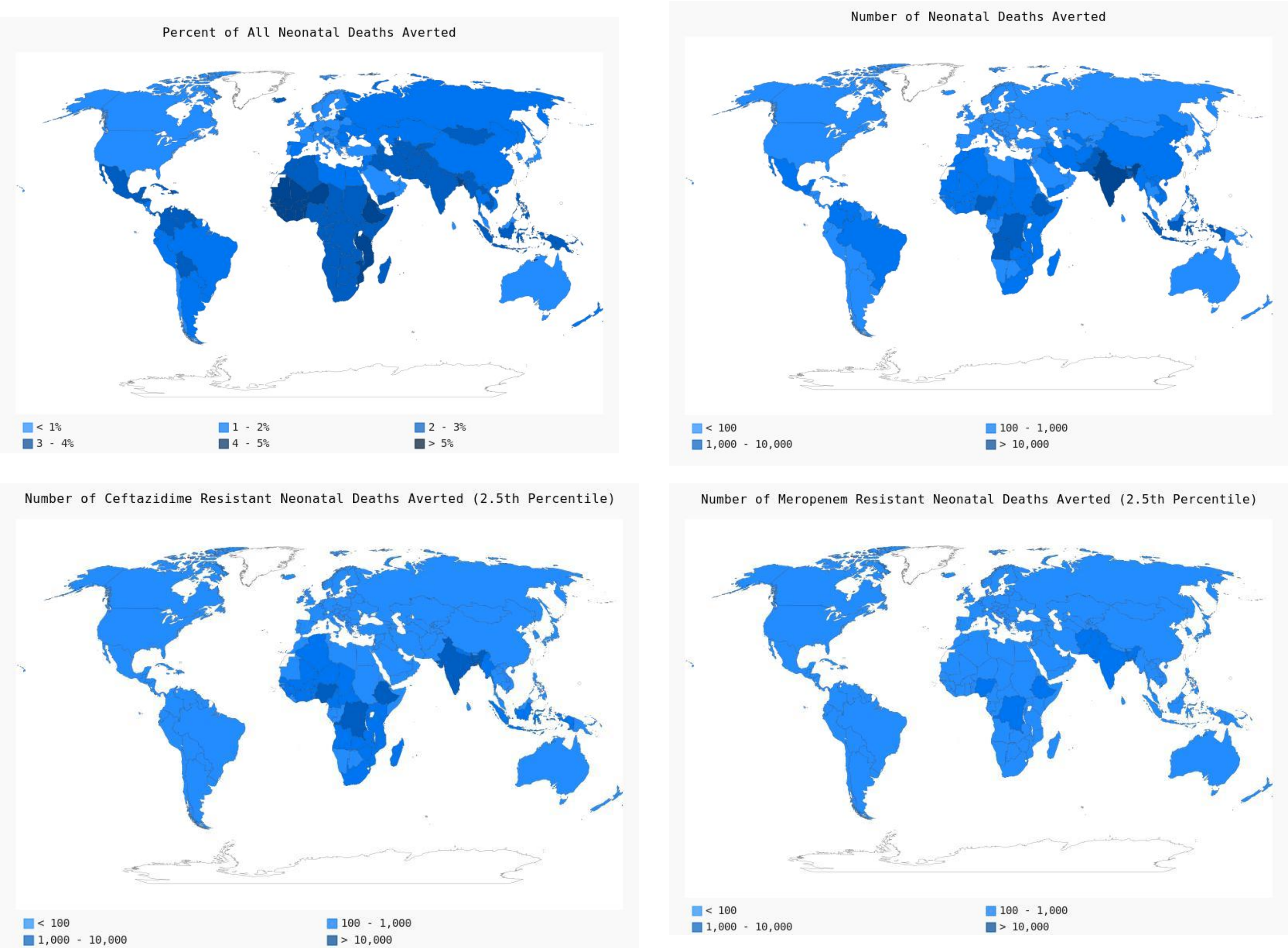

Figure H. 2.5th percentile (i.e., lower bound) of estimates represented as maps shown in Fig. 3. The maps are reprinted from pygal_maps_world under GNU GPL.

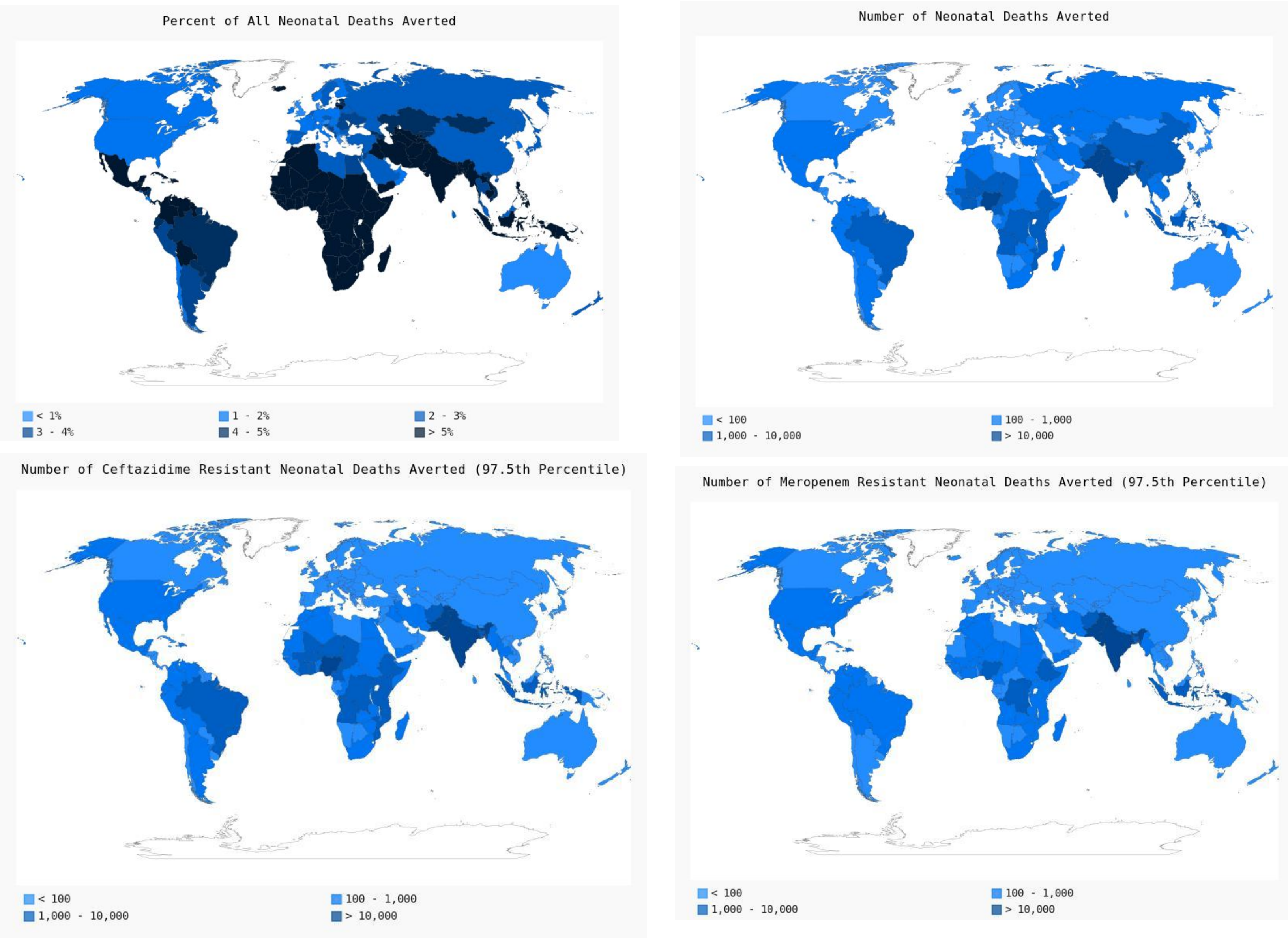

Figure I. 97.5th percentile (i.e., lower bound) of estimates represented as maps shown in Fig. 3. The maps are reprinted from pygal_maps_world under GNU GPL.

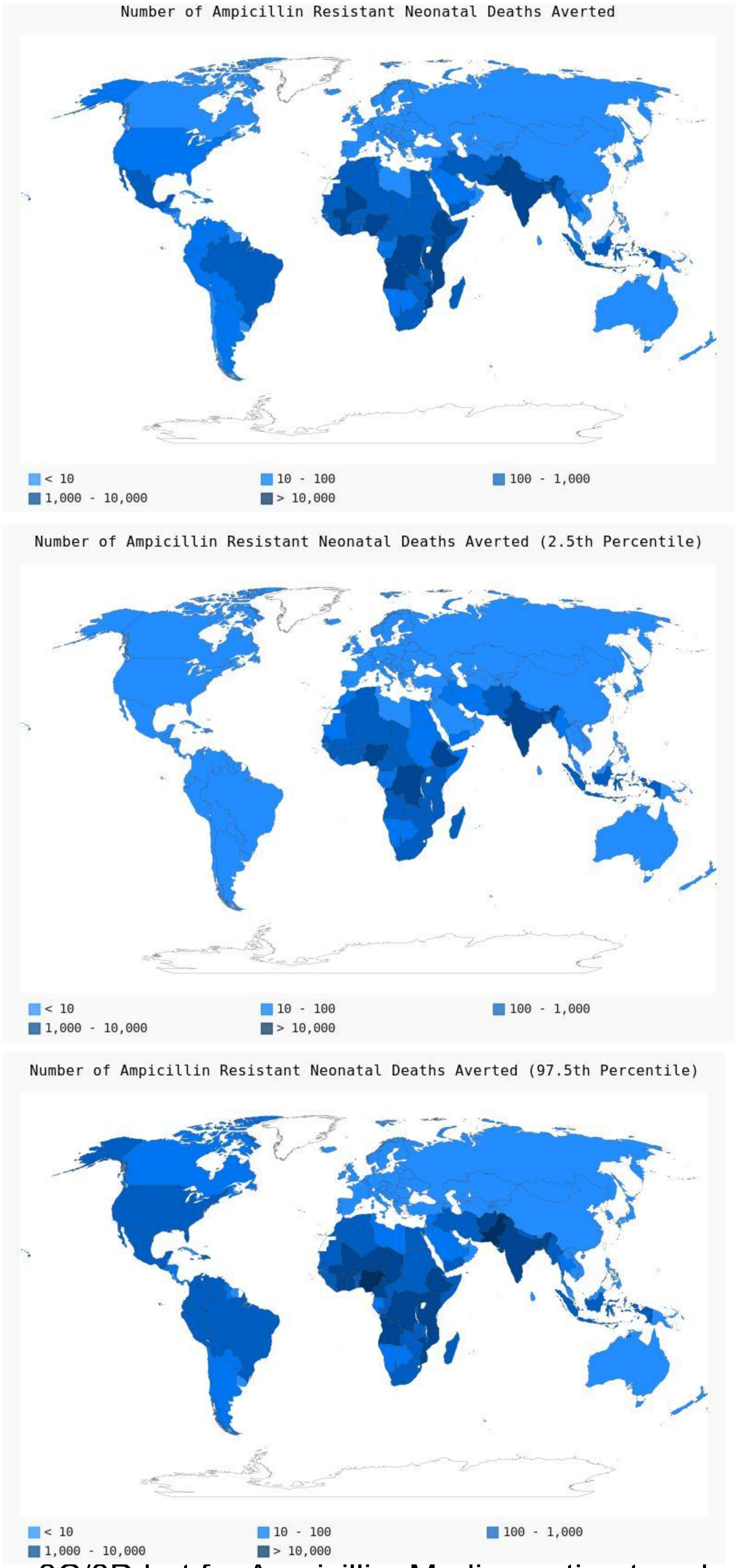

Figure J. As. Fig. 3C/3D but for Ampicillin. Median estimates shown on top. 2.5th percentile shown in middle. 97.5th percentile shown on bottom. The maps are reprinted from pygal_maps_world under GNU GPL.

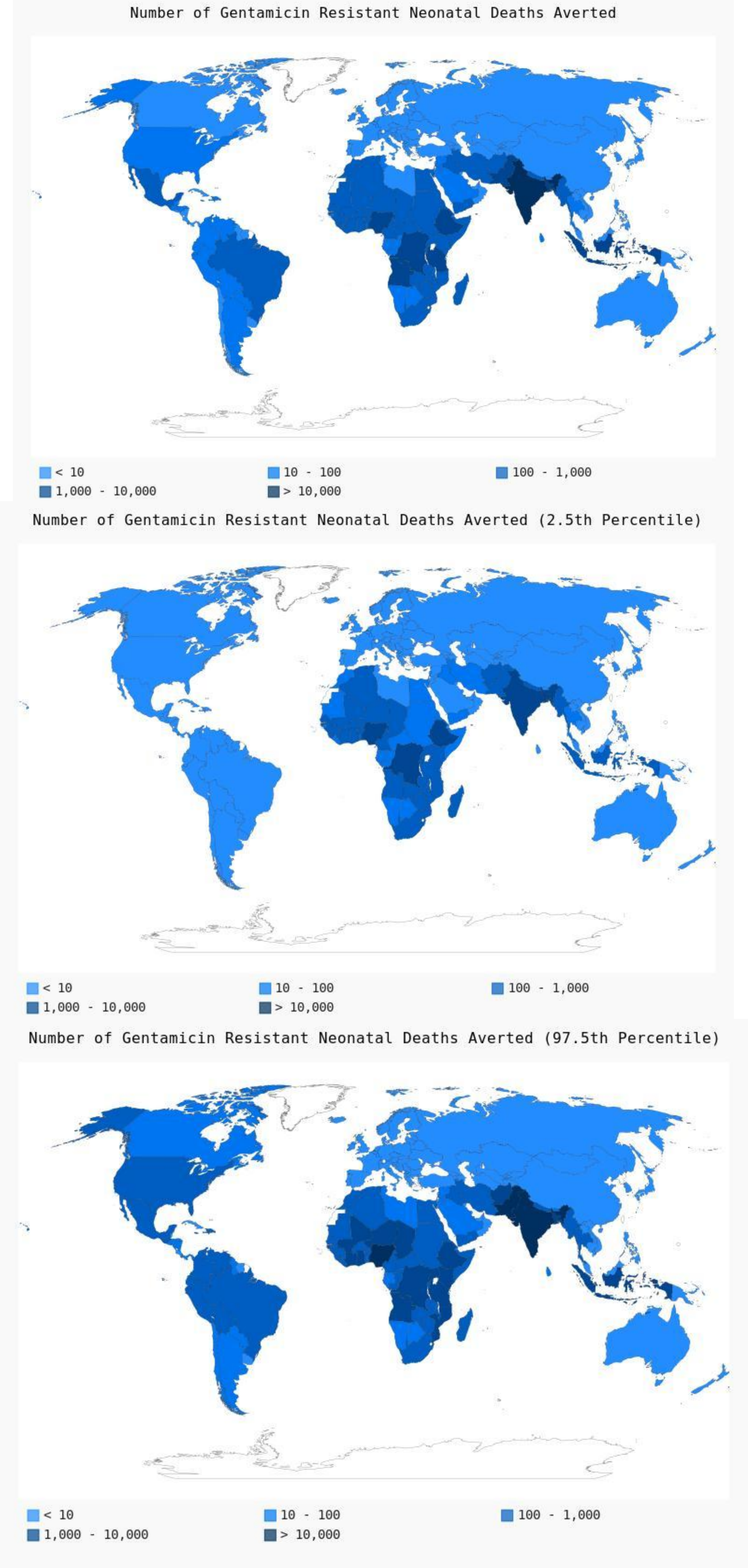

Figure K. As. Fig. 3C/3D but for Gentamicin. Median estimates shown on top. 2.5th percentile shown in middle. 97.5th percentile shown on bottom. The maps are reprinted from pygal_maps_world under GNU GPL.

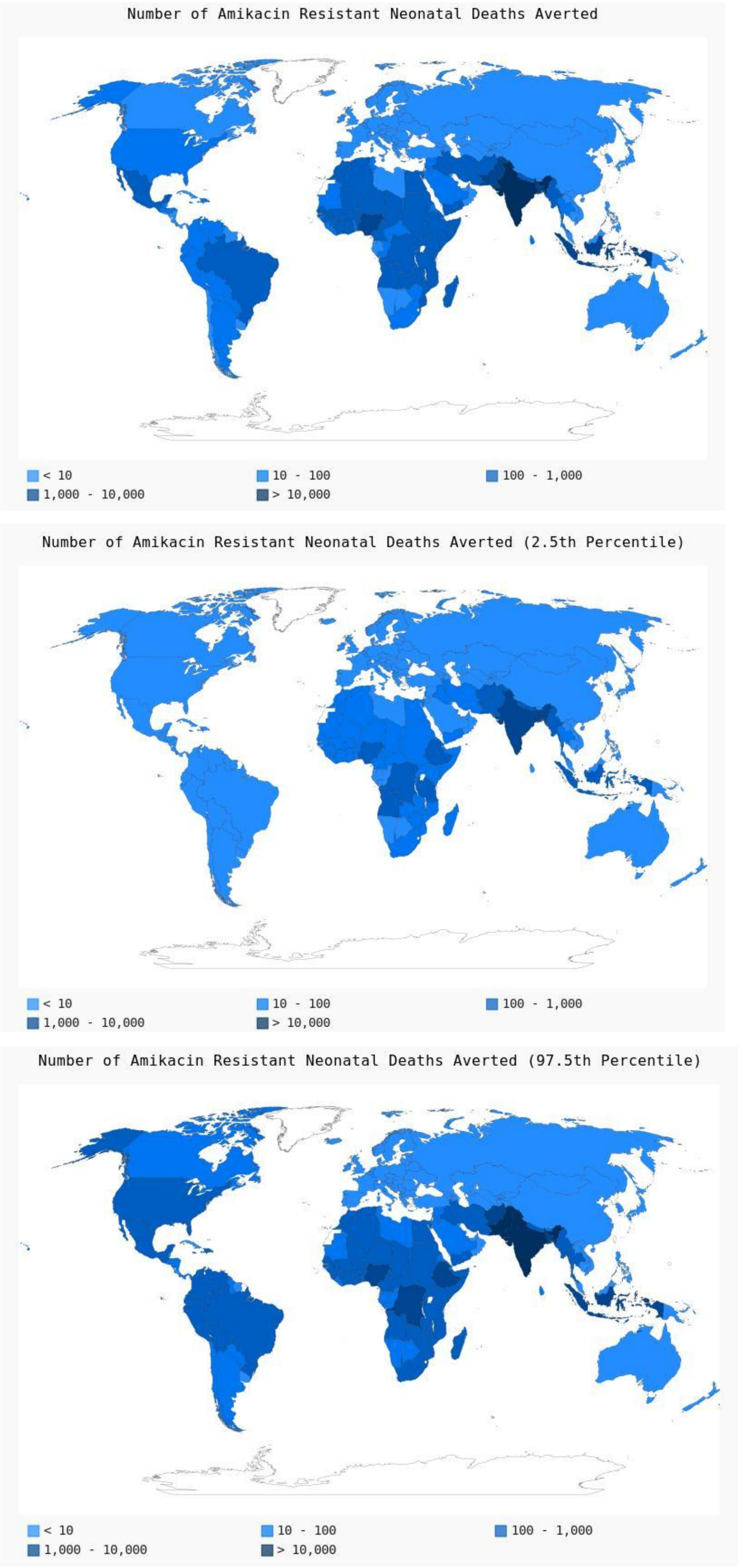

Figure L. As. Fig. 3C/3D but for Amikacin. Median estimates shown on top. 2.5th percentile shown in middle. 97.5th percentile shown on bottom. The maps are reprinted from pygal_maps_world under GNU GPL.

Dependent Variable	Independent Variable	Coefficient (standard error): t-value [95%tile Confidence Interval	Constant (standard error): t-value [95%tile Confidence Interval	R^2	F-statistic (p-value)
Number of unique carbapenamase resistant genes per isolate	Year	0.0497 (0.004): 13.167 [0.042 - 0.058]	-99.61 (7.59): -13.12 [-115.62 - -83.56]	0.911	173.4 (2.40e-10)
Number of unique aminoglycoside resistant genes per isolate	Year from 2003 onwards	0.0117 (0.801): 0.801 [-0.019 - 0.043]	-20.771 (29.29): -0.71 [-83.21 - 41.65]	0.041	0.6417 (0.436)

Table D. Regression analysis results for the model used to estimate the yearly rate of increase in antimicrobial resistance genes.

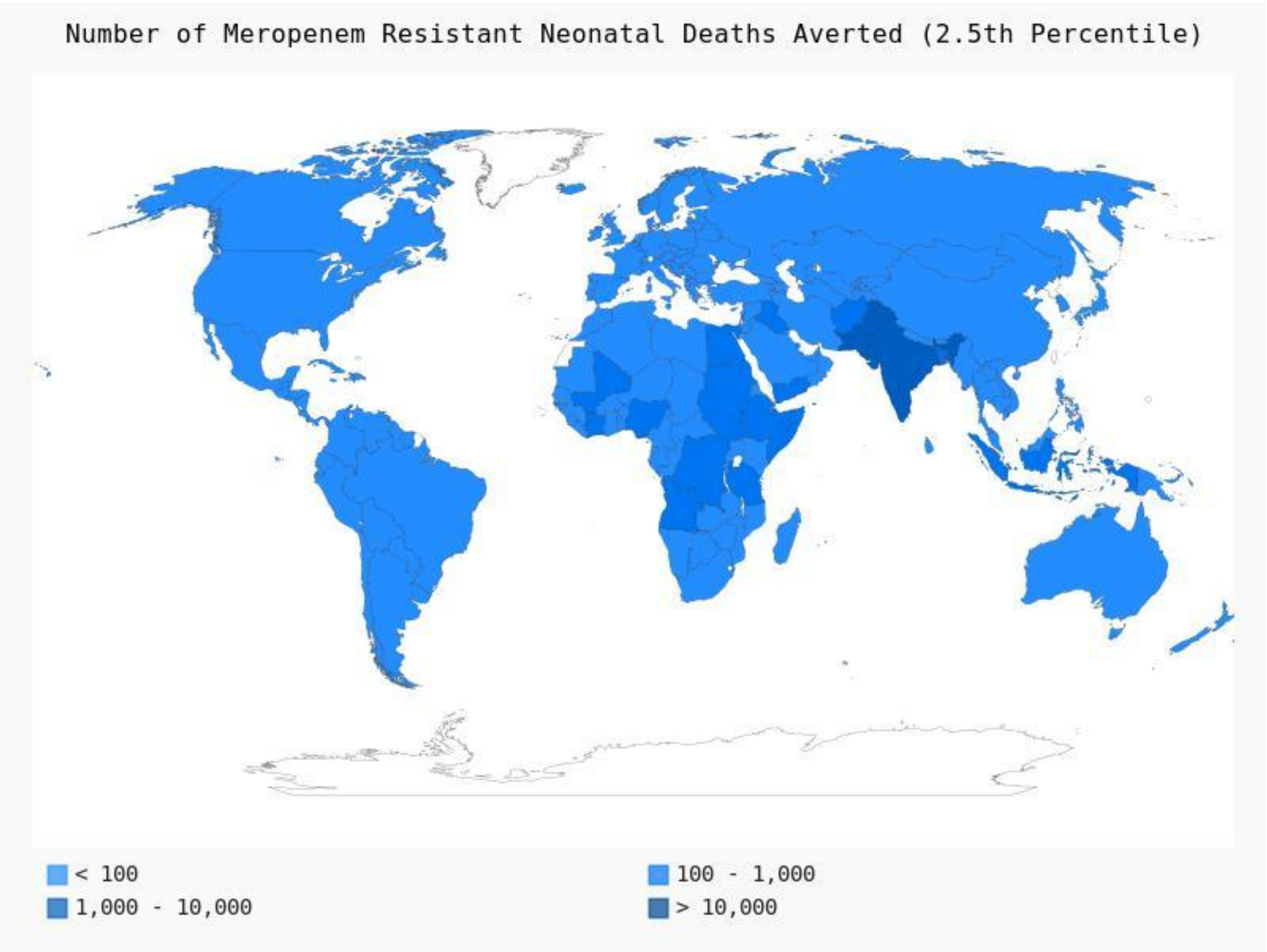

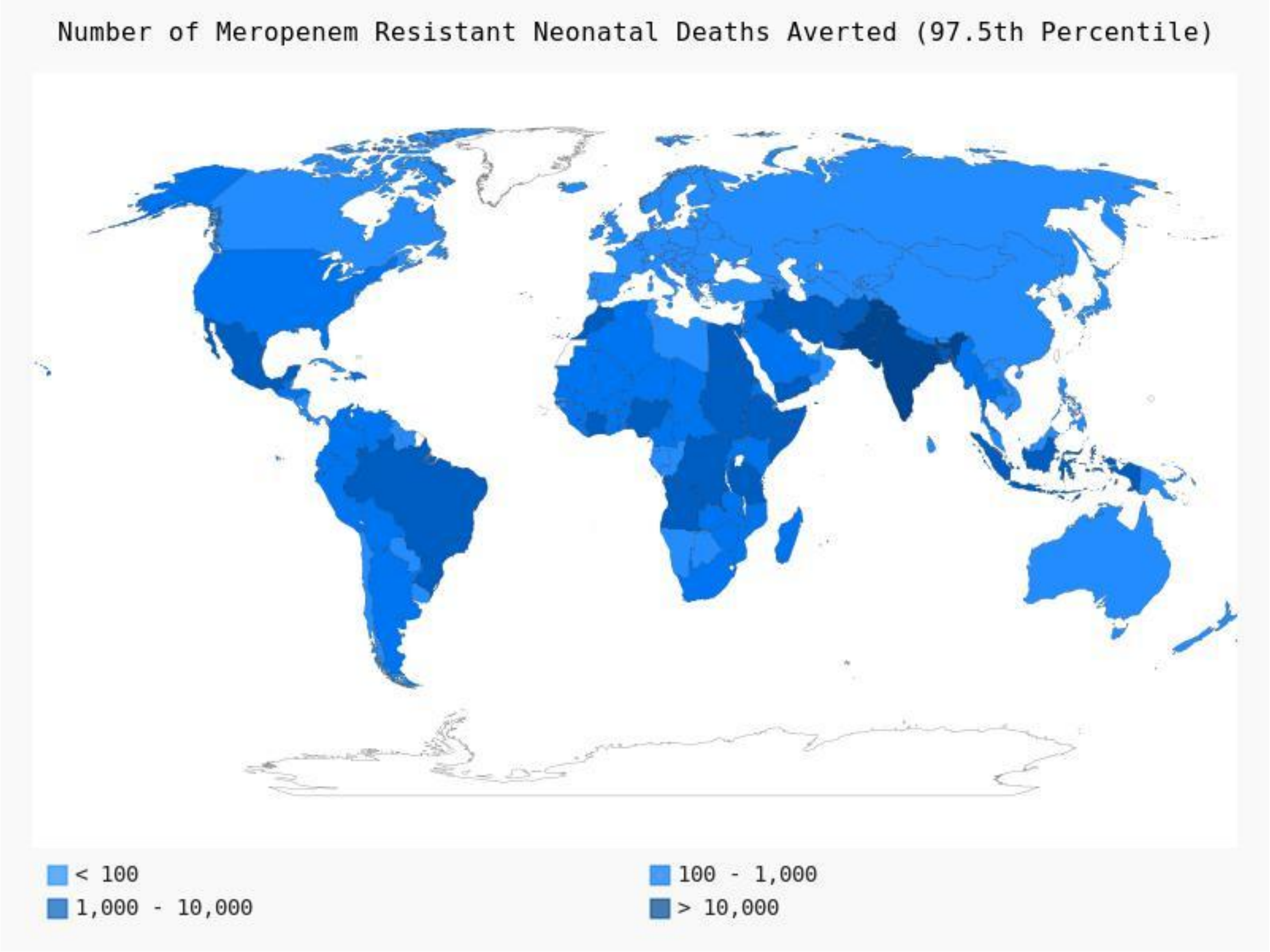

Figure M. Credible interval of map shown in Fig. 4B. 2.5th percentile shown on top and 97.5th percentile shown on bottom. The maps are reprinted from pygal_maps_world under GNU GPL.

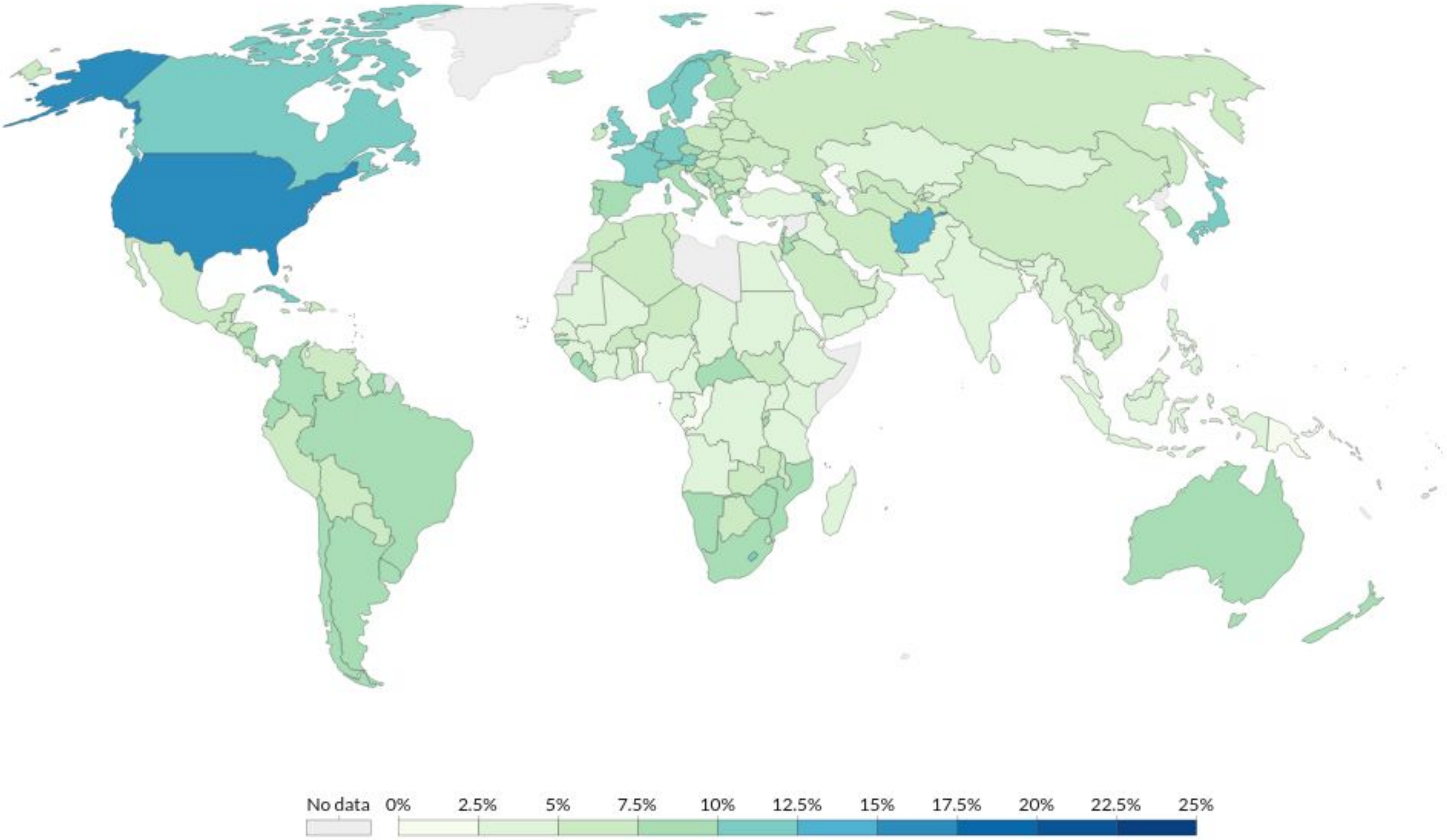

Figure N. Health expenditure as a fraction of the country's GDP. Data courtesy of the WHO, Global Health Observatory (2022). Map reprinted from OurWorldInData under a CC-BY license. Original: https://ourworldindata.org/grapher/total-healthcare-expenditure-gdp.

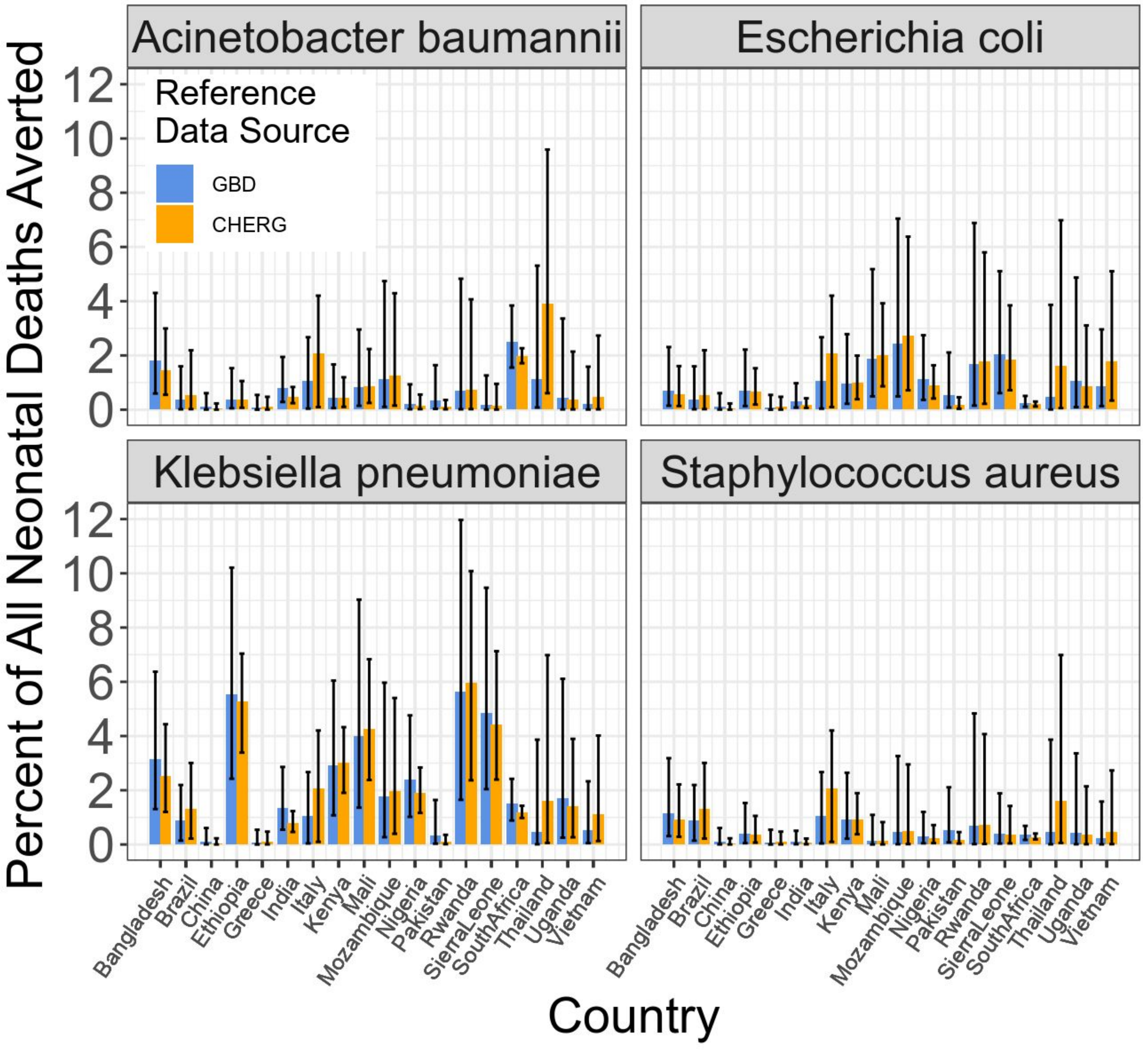

Figure O. As Fig. 1A but for other etiologies of interest. Median estimated fraction of neonatal deaths averted given maternal vaccination against a specific pathogen at 70% efficacy and coverage equivalent to that of the maternal tetanus vaccine. Median shown; error bars indicate 95th percentile Bayesian credible intervals. GBD refers to data from the Global Burden of Disease study, and CHERG refers to data from the Child Health and Epidemiology Reference Group.

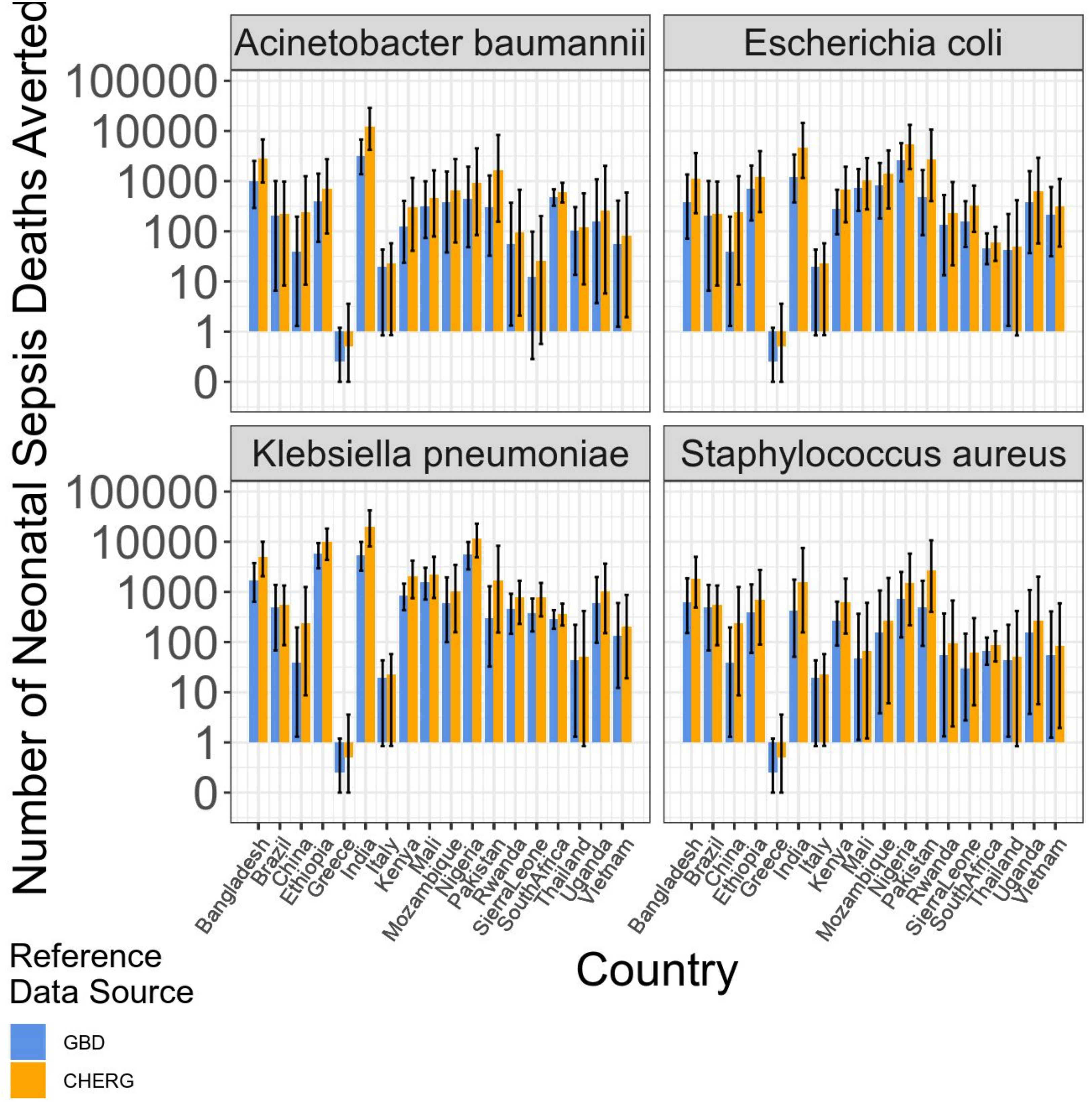

Figure P. As Fig. 1B but for other etiologies of interest. Median estimated number of avertable neonatal sepsis deaths given maternal vaccination against a specific pathogen at 70% efficacy and coverage equivalent to that of the maternal tetanus vaccine. Median shown; error bars indicate 95th percentile Bayesian credible intervals. A pseudo log transform is done for values between zero and one. GBD refers to data from the Global Burden of Disease study, and CHERG refers to data from the Child Health and Epidemiology Reference Group.

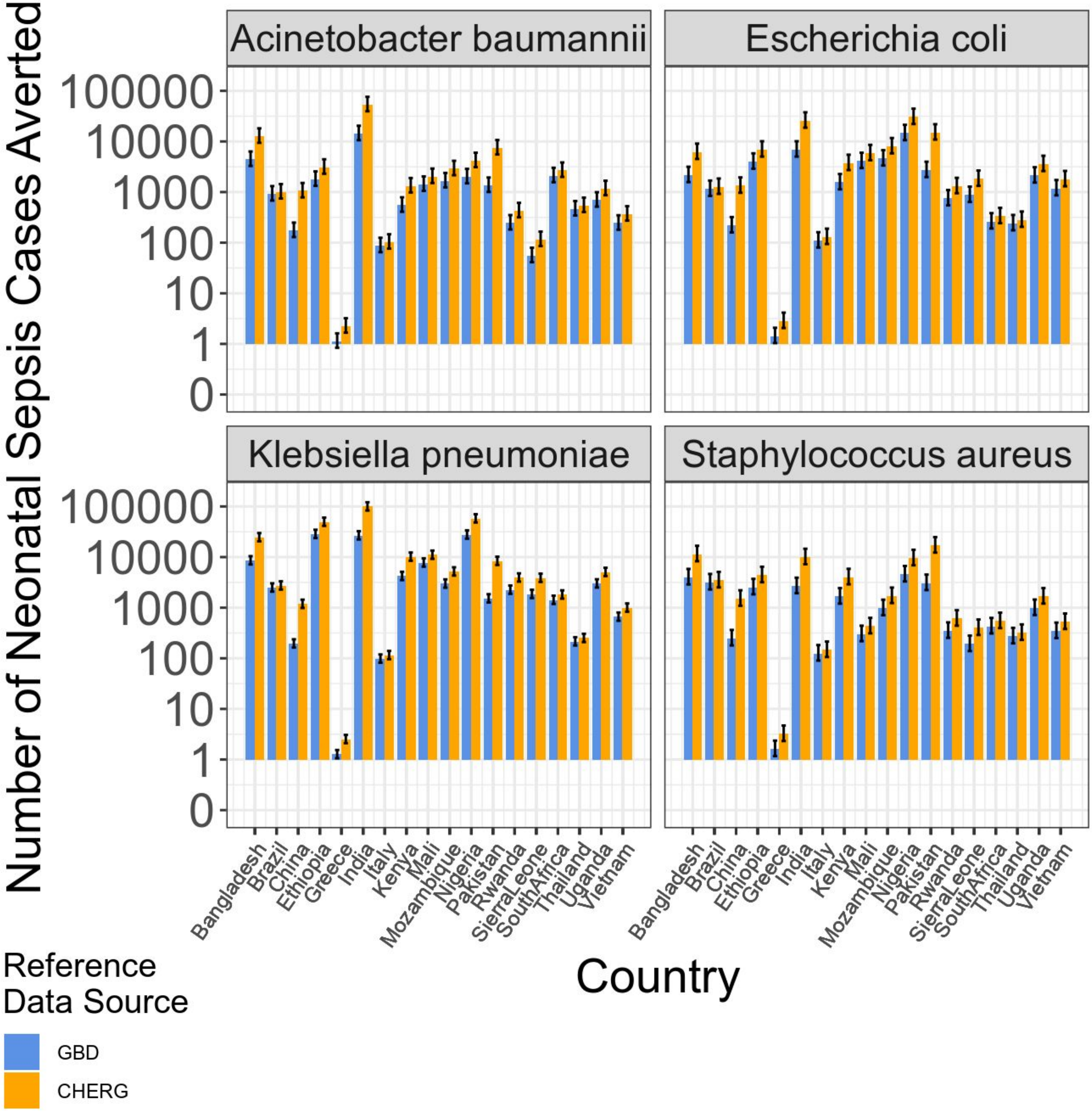

Figure Q. As Fig. 1C but for other etiologies of interest. Median estimated number of avertable neonatal sepsis cases given maternal vaccination against a specific pathogen at 70% efficacy and coverage equivalent to that of the maternal tetanus vaccine. Median shown; error bars indicate 95th percentile Bayesian credible intervals. GBD refers to data from the Global Burden of Disease study, and CHERG refers to data from the Child Health and Epidemiology Reference Group.

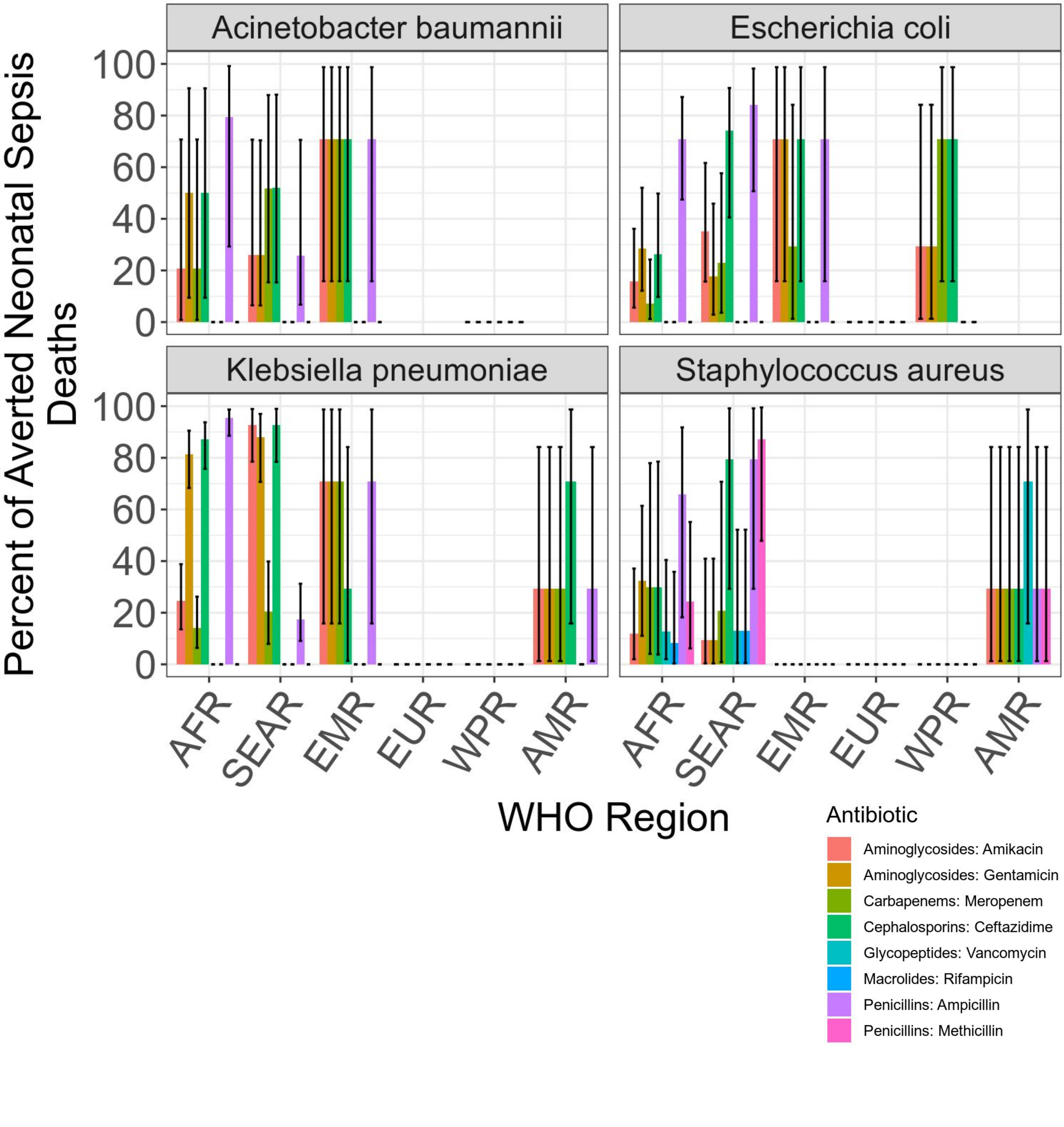

Figure R. As Fig. 2A but for other etiologies and relevant antibiotics of interest. Estimated median fraction of isolates from neonates who died with culture-confirmed sepsis that are resistant to various drugs across WHO regions. Median shown; error bars indicate 95th percentile Bayesian credible intervals.

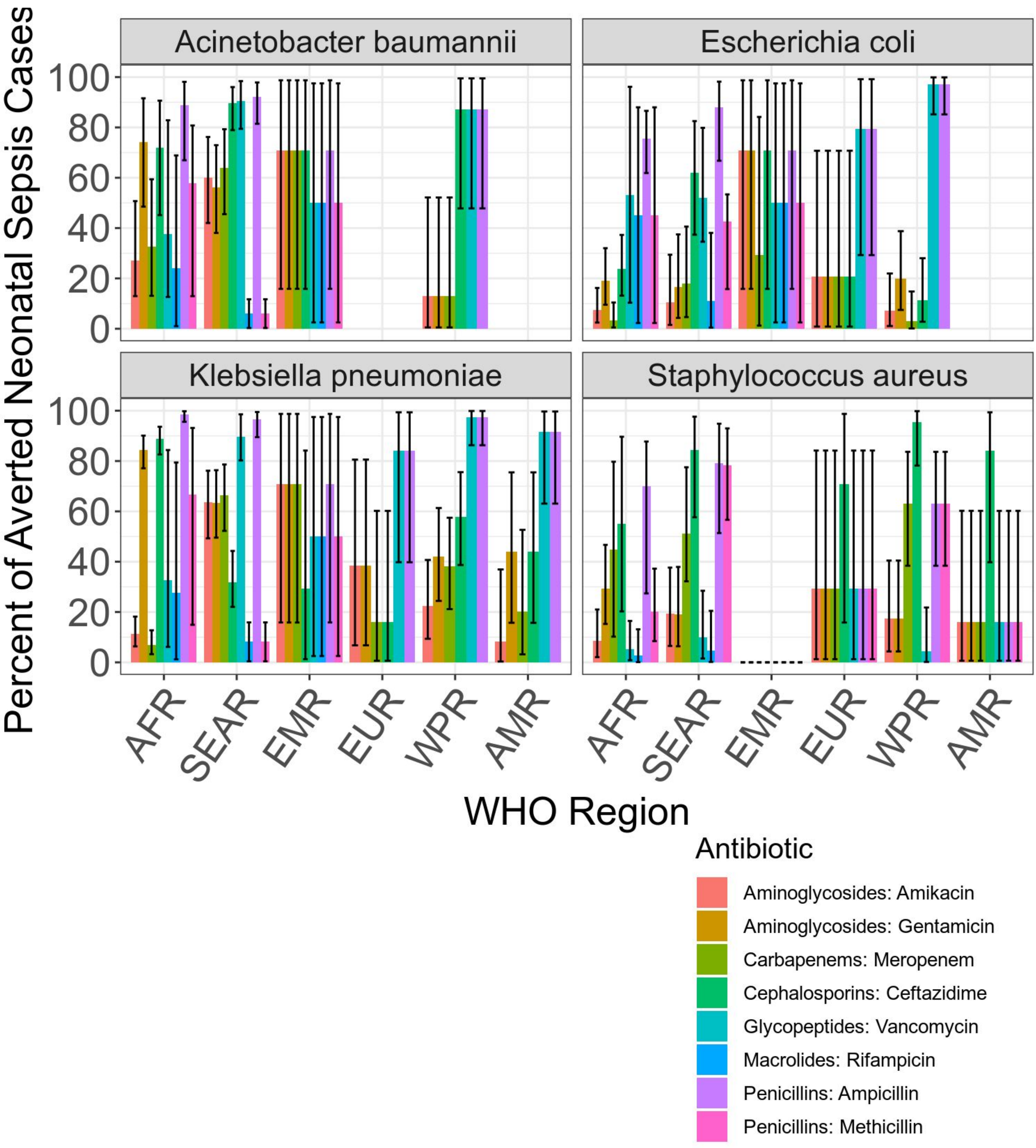

Figure S. As Fig. 2B but for other etiologies and relevant antibiotics of interest. Estimated median fraction of isolates from neonates with culture-confirmed sepsis that are resistant to various drugs across WHO regions. Median shown; error bars indicate 95th percentile Bayesian credible intervals.

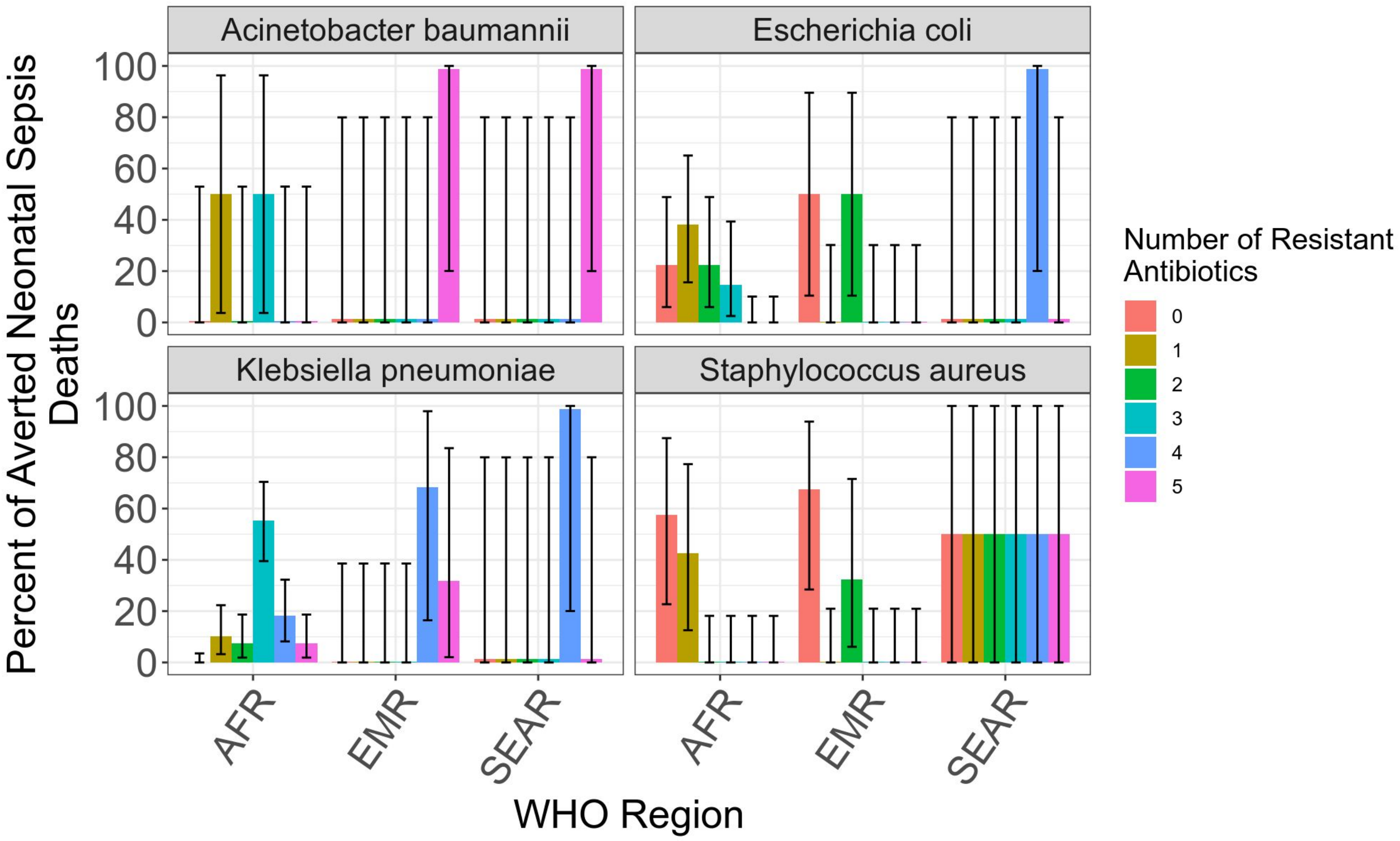

Figure T. As Fig. 2E but for other etiologies of interest. Antibiotics considered are shown in Fig. R and S in S1 Text. Median shown; error bars indicate 95th percentile Bayesian credible intervals.

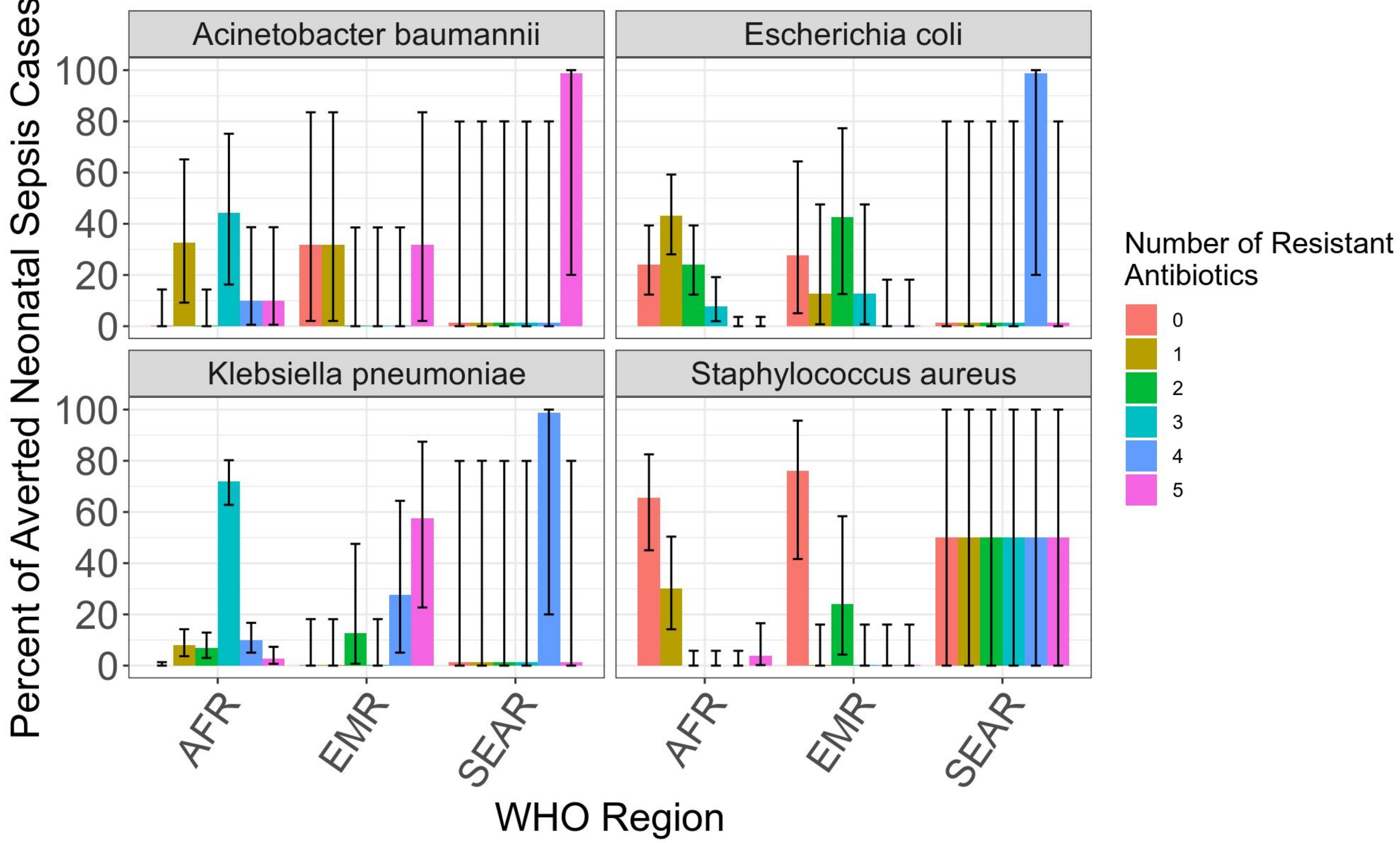

Figure U. As Fig. 2F but for other etiologies of interest. Antibiotics considered are shown in Fig. R and S in S1 Text. Median shown; error bars indicate 95th percentile Bayesian credible intervals.
