# Supplementary material for: Global, regional, and national estimates of the impact of a maternal Klebsiella pneumoniae vaccine: A Bayesian modeling analysis
Source: PLoS Med. 2023 May 22;20(5):e1004239. doi: 10.1371/journal.pmed.1004239 (PMC10270628; doi:10.1371/journal.pmed.1004239)
Supplement: S2 Text — Derivation of the mixture model posterior distribution (Section 1) and details of antimicrobial susceptibility testing done (Section 2). (PDF) [file pmed.1004239.s002.pdf]

Global, regional, and national estimates of the impact of a maternal *Klebsiella pneumoniae* vaccine: A Bayesian modeling analysis

S2 Text: Extended Methods

Chirag K. Kumar[1], Kirsty Sands[2], Timothy R. Walsh[2], Seamus O'Brien[3], Mike Sharland[4], Joseph A. Lewnard[5], Hao Hu[6], Padmini Srikantiah[6], Ramanan Laxminarayan[1, 7*]

1 Princeton University, Princeton, New Jersey, USA

2 Ineos Oxford Institute for Antimicrobial Resistance, Department of Zoology, Oxford, UK

3 Global Antibiotic Research and Development Partnership, Geneva, Switzerland

4 Center for Neonatal and Paediatric Infection (CNPI), Institute of Infection and Immunity, St George's University of London, London, UK

5 Division of Epidemiology, School of Public Health, University of California at Berkeley, Berkeley, CA

6 Bill & Melinda Gates Foundation, Seattle, Washington, USA

7 One Health Trust, Bengaluru, India

* ramanan@onehealthtrust.org

Section 1.1: Derivation of the Posterior Distribution

We analytically derive the posterior probability distribution for the quantity $p_{s,l}$ referenced in the main text. We abbreviate this quantity as p as the derivation is identical for specific s or l. We show that the posterior follows a standard univariate beta distribution. First, let us write the likelihood function for observing N_{kp} deaths out of N_{d} deaths in total sampled by a given study at a given location:

$$\mathcal{L}(N_{\mathrm{d}}, N_{\mathrm{kp}} \mid p) = \binom{N_{\mathrm{d}}}{N_{\mathrm{kp}}} p^{N_{\mathrm{kp}}}(1-p)^{N_{\mathrm{d}}-N_{\mathrm{kp}}} \tag{1}$$

where $\binom{\cdot}{\cdot}$ is the choose function.

We seek to determine the probability distribution of p, or in other words to determine the density function $f(p|N_{\mathrm{d}}, N_{\mathrm{kp}})$. We apply Bayes' theorem:

$$f(p|N_{\mathrm{d}}, N_{\mathrm{kp}}) = \frac{\mathcal{L}(N_{\mathrm{d}}, N_{\mathrm{kp}} \mid p) f(p)}{\int_p \mathcal{L}(N_{\mathrm{d}}, N_{\mathrm{kp}} \mid p) f(p) \mathrm{d}p} \tag{2}$$

where $f(p)$ is the prior probability distribution on p. We write the prior on p as a beta distribution with shape parameters a and b, $\beta(p|a,b) = \frac{1}{B(a,b)} p^{a-1} p^{b-1}$ since p is a probability.

$$f(p|N_{\mathrm{d}}, N_{\mathrm{kp}}) = \frac{\binom{N_{\mathrm{d}}}{N_{\mathrm{kp}}} p^{N_{\mathrm{kp}}}(1-p)^{N_{\mathrm{d}}-N_{\mathrm{kp}}} \frac{p^{a-1}(1-p)^{b-1}}{B(a,b)}}{\int_0^1 \binom{N_{\mathrm{d}}}{N_{\mathrm{kp}}} p^{N_{\mathrm{kp}}}(1-p)^{N_{\mathrm{d}}-N_{\mathrm{kp}}} \frac{p^{a-1}(1-p)^{b-1}}{B(a,b)} \mathrm{d}p} \tag{3}$$

where $B(a,b) = \frac{\Gamma(a)\Gamma(b)}{\Gamma(a+b)}$ and $\Gamma(\cdot)$ is the Gamma function.

$$f(p|N_{\mathrm{d}}, N_{\mathrm{kp}}) = \frac{\frac{\binom{N_{\mathrm{d}}}{N_{\mathrm{kp}}}}{B(a,b)} p^{N_{\mathrm{kp}}}(1-p)^{N_{\mathrm{d}}-N_{\mathrm{kp}}} p^{a-1}(1-p)^{b-1}}{\frac{\binom{N_{\mathrm{d}}}{N_{\mathrm{kp}}}}{B(a,b)} \int_0^1 p^{N_{\mathrm{kp}}}(1-p)^{N_{\mathrm{d}}-N_{\mathrm{kp}}} p^{a-1}(1-p)^{b-1} \mathrm{d}p} \tag{4}$$

$$f(p|N_{\mathrm{d}}, N_{\mathrm{kp}}) = \frac{p^{N_{\mathrm{kp}}+a-1}(1-p)^{N_{\mathrm{d}}-N_{\mathrm{kp}}+b-1}}{\int_0^1 p^{N_{\mathrm{kp}}+a-1}(1-p)^{N_{\mathrm{d}}-N_{\mathrm{kp}}+b-1} \mathrm{d}p} \tag{5}$$

Note how the integrand in the denominator is the beta function, $B(N_{\mathrm{kp}}+a, N_{\mathrm{d}}-N_{\mathrm{kp}}+b)$. Beyond noting this definition, this can also be seen by observing that the fraction equals the density of a beta distribution divided by an integrand in dp that must be a normalizing factor so that the probability density integrates to one over the entire domain on p. The normalizing factor for a beta distribution is a beta function, in this case $B(N_{\mathrm{kp}}+a, N_{\mathrm{d}}-N_{\mathrm{kp}}+b)$.

$$f(p|N_{\mathrm{d}}, N_{\mathrm{kp}}) = \frac{p^{N_{\mathrm{kp}}+a-1}(1-p)^{N_{\mathrm{d}}-N_{\mathrm{kp}}+b-1}}{B(N_{\mathrm{kp}}+a, N_{\mathrm{d}}-N_{\mathrm{kp}}+b)} \tag{6}$$

This is the density for a beta distribution.

$$f(p|N_{\mathrm{d}}, N_{\mathrm{kp}}) = \beta(p|N_{\mathrm{d}}+a, N_{\mathrm{d}}-N_{\mathrm{kp}}+b) = \frac{p^{N_{\mathrm{kp}}+a-1}(1-p)^{N_{\mathrm{d}}-N_{\mathrm{kp}}+b-1}}{B(N_{\mathrm{kp}}+a, N_{\mathrm{d}}-N_{\mathrm{kp}}+b)} \tag{7}$$

In our case, the prior on p is a uniform distribution, so $a = b = 1$ ($\beta(p|a=1, b=1) = 1$):

$$f(p|N_{\mathrm{d}}, N_{\mathrm{kp}}) = \beta(p|N_{\mathrm{d}}+1, N_{\mathrm{d}}-N_{\mathrm{kp}}+1) = \frac{p^{N_{\mathrm{kp}}}(1-p)^{N_{\mathrm{d}}-N_{\mathrm{kp}}}}{B(N_{\mathrm{kp}}+1, N_{\mathrm{d}}-N_{\mathrm{kp}}+1)} \tag{8}$$

Written as a sampling statement:

$$p \sim \beta(N_{\mathrm{kp}}+1, N_{\mathrm{d}}-N_{\mathrm{kp}}+1) \tag{9}$$

Section 1.2: AMR Susceptibility Testing

In data gathered through the Burden of Antibiotic Resistance in Neonates from Developing Societies (BARNARDS), isolated bacterial cultures from blood samples of those neonates with culture-confirmed sepsis were tested for antibiotic susceptibility using agar dilution to calculate the minimum inhibitory concentration against various antibiotics used to treat sepsis. In data gathered through the Global Neonatal Sepsis Observational Study (NeoObs), the local technique used by the lab site gathering the isolates was used. In both studies, European Committee on Antimicrobial Susceptibility Testing (EUCAST) guidelines were used to interpret the observed minimum inhibitory concentration of the isolate against an antibiotic and classify the sample as resistant, intermediate, or susceptible.

For data gathered through BARNARDS, we had access to the susceptibility testing at the isolate level, so we could determine all the antibiotics to which a single isolate was resistant. This enabled us to evaluate the multi-drug resistance of the isolate in World Health Organization (WHO) regions from which BARNARDS data was gathered (Africa, South-East Asia, and the Eastern Mediterranean).
