# Supplementary material for: Global, regional, and national estimates of the impact of a maternal Klebsiella pneumoniae vaccine: A Bayesian modeling analysis
Source: PLoS Med. 2023 May 22;20(5):e1004239. doi: 10.1371/journal.pmed.1004239 (PMC10270628; doi:10.1371/journal.pmed.1004239)
Supplement: Attachment — Submitted filename: ResponseToReviewers-R1-02152023.docx [file pmed.1004239.s005.docx]

February 15^th^, 2023

PLOS Medicine

Dear Dr. Dobbs,

We thank you and the reviewers for the detailed comments on our manuscript, *Global, regional, and national estimates of the impact of a maternal Klebsiella pneumoniae vaccine: A Bayesian modeling analysis*. We have largely incorporated the suggestions and believe that they have helped to improve the manuscript. Major changes are summarized below; a point-by-point response to all queries is also attached.

At the suggestions of the reviewers and the editors, we have added text discussing the data used for our analysis to the Abstract and Methods section. In particular, we have included details on the size of the datasets and an additional table that describes the nature of *K. pneumoniae* surveillance within each country contributing data to the analysis. Furthermore, we have edited the figures to improve interpretability, and have added detail to the Methods section on (a) the extrapolation procedure used to go from 18 countries to all countries and (b) the analysis of *K. pneumoniae* genomes to identify trends in antimicrobial resistance gene acquisition.

After consideration of Reviewer #1’s concerns about our extrapolations from the 18 countries for which we have surveillance data to global estimates of averted neonatal sepsis, we have limited our extrapolations in the main text to only low- and middle-income countries as the 18 countries for which we have data are mainly low- and middle-income countries. We have highlighted our assessment of the model goodness-of-fit to justify why this approach is reasonable and the use of our Bayesian mixture model to provide meaningful estimates despite uncertainty in the underlying data. We also expand our discussion of limitations in our approach.

In the revision, we include sensitivity analyses of key parameters, tables detailing model parameters, and other measures that we hope improve the transparency and reproducibility of our work.

We thank the editorial team and reviewers again for their assessment of the manuscript and thoughtful suggestions. We look forward to your decision.

Sincerely,

Ramanan Laxminarayan

Dr. Ramanan Laxminarayan and co-authors

Requests from the editors:

GENERAL
Please respond to all editor and reviewer requests detailed below, in full.
Please include line numbers starting at 1 on the title page and in continuous sequence thereafter

E.1) COMPETING INTERESTS STATEMENT
“The sponsor of the study provided input in the writing of the report, acting in a purely scientific capacity.” Please elaborate further on the precise role that the funder played in writing the manuscript.  What does “purely scientific capacity” encompass?  Please include details of each individual affiliated to the funding body that contributed to manuscript and provide details of each individual’s specific role in the preparation of the manuscript.

The two authors from the Bill & Melinda Gates Foundation provided their expertise on *K. pneumoniae* epidemiology and vaccine development. There is little expertise easily accessible to us, particularly on this latter aspect. Dr. Padmini Srikantiah is an infectious disease epidemiologist who has worked extensively on neonatal sepsis and understands the CHAMPS, NeoObs, and BARNARDS data sets well, which we use in our modeling analyses. Hao Hu is a disease modeler who has worked extensively on the modeling of bacterial pathogens, including those causing neonatal sepsis, and his scientific inputs into our model were particularly helpful. We have revised the competing interests statement as follows:

“Two study authors (PS and HH) are employed by the organization that funded the study. They provided input in the writing of the report, acting in a purely scientific capacity. In particular, PS provided critical expertise on *K. pneumoniae* epidemiology and vaccine development. HH provided valuable insights into modeling bacterial pathogens.”

E.2) DATA AVAILABILITY STATEMENT
Thank you for agreeing to make your data available upon request.  The Data Availability Statement (DAS) requires revision. For each data source used in your study:
a) If the data are freely or publicly available, note this and state the location of the data: within the paper, in Supporting Information files, or in a public repository (include the DOI or accession number).
b) If the data are owned by a third party but freely available upon request, please note this and state the owner of the data set and contact information for data requests (web or email address). Note that a study author cannot be the contact person for the data.
c) If the data are not freely available, please describe briefly the ethical, legal, or contractual restriction that prevents you from sharing it. Please also include an appropriate contact (web or email address) for inquiries (again, this cannot be a study author).

Please see here a revised data accessibility statement:

“The CHAMPS data are freely available online upon request. In this analysis, we used CHAMPS Level 2: De-Identified Data, which are all available at the following link after signing a data transfer agreement: <https://champshealth.org/data/>.

BARNARDS data are owned by the Ineos Oxford Institute for Antimicrobial Resistance. They are freely available upon request from the principal investigators of the study. For details, please reach out to Kathryn Thomson at [kathryn.thomson@zoo.ox.ac.uk](mailto:kathryn.thomson@zoo.ox.ac.uk). NeoObs data are owned by the Global Antibiotic Research and Development Partnership and are freely available upon request. For details, please contact Sally Ellis at [sellis@gardp.org](mailto:sellis@gardp.org).

The genomes analyzed in this paper are all available through the European Nucleotide Archive under project number PRJEB33565. Individual accession numbers for all genomes analyzed are also available as part of Table S2.”

E.3) RESEARCH IN CONTEXT
Please remove this section and all sub-sections from the manuscript

We have made the requested change in the revised text.

E.4) Please structure your abstract using the PLOS Medicine headings (Background, Methods and Findings, Conclusions). Please combine the Methods and Findings sections into one section, “Methods and findings”.

We have made the requested change in the revised text.

Abstract Background:
E.5) Please ensure that the final sentence clearly states the study question.

We have made the requested change in the revised text and the new sentence reads as follows (lines 26 to 29):

“We estimated the potential impact of such vaccination on cases and deaths of *K. pneumoniae* neonatal sepsis and project the global effects of routine immunization of pregnant women with the *K. pneumoniae* vaccine as antimicrobial resistance increases.”

E.6) “…resistant to antibiotic treatment regimens…” suggest introducing carbapenem resistance here at the outset, rather than later

We have made the requested change in the revised text and the new sentence reads as follows (lines 20 to 24):

“*Klebsiella pneumoniae* (*K. pneumoniae*) is the leading pathogen globally underlying cases of neonatal sepsis, and is frequently resistant to antibiotic treatment regimens recommended by the World Health Organization (WHO), including first-line therapy with ampicillin and gentamicin, second-line therapy with amikacin and ceftazidime, and meropenem.”

E.7) Please define “K. pneumoniae” after the full name in the lines above, prior to first use here

We have made the requested change in the revised text (line 20).

Abstract Methods and Findings:
E.8) “We used data from three global studies…” It would be helpful to include further details here - which countries did those studies include, how many participants, over what time frame (s) how many had K. pneumoniae and so on

We have included further details of the data including the requested information and the new sentence reads as follows (lines 30 to 39):

“To parameterize our model, we used data from three global studies of neonatal sepsis and/or mortality – with 2,330 neonates who died with sepsis surveilled from 2016 to 2020, undertaken in 18 mainly low- and middle-Income countries across all WHO regions (Ethiopia, Kenya, Mali, Mozambique, Nigeria, Rwanda, Sierra Leone, South Africa, Uganda, Brazil, Italy, Greece, Pakistan, Bangladesh, India, Thailand, China, and Vietnam). Within these studies, 26.95% of fatal neonatal sepsis cases were culture-positive for *K. pneumoniae*.”

Additionally, we have built upon this comment to add a better description of the underlying datasets in the data subsection of the Methods section (lines 145 to 163):

“Three data sets were used to quantify *K. pneumoniae* neonatal sepsis burden: the Child Health and Mortality Prevention Surveillance (CHAMPS),^4^ the Burden of Antibiotic Resistance in Neonates from Developing Societies (BARNARDS),^5^ and the Global Neonatal Sepsis Observational Study (NeoObs).^26^ CHAMPS, initiated in 2016, conducts autopsies on children under five to determine causal drivers of death at eight study sites in seven low- and middle-income countries (LMICs) across Africa and South-East Asia (Bangladesh, two sites in Ethiopia, Kenya, Mali, Mozambique, Sierra Leone, and South Africa). In contrast, BARNARDS and NeoObs are birth-cohort studies that tracked neonates prospectively through their first month of life, evaluating incidence of sepsis, the antibiotic susceptibility of associated isolates, and resulting mortality. BARNARDS surveilled neonates across 12 study sites in seven countries in Africa and South-East Asia (two sites in Bangladesh, one in India, two in Pakistan, Ethiopia, three in Nigeria, two in Rwanda, and one in South Africa) from 2015 to 2017. Finally, NeoObs reported neonatal sepsis cases, deaths, and antibiotic susceptibility of the isolated bacteria in 19 sites across 11 countries in Europe (Italy, Greece), Latin America (two sites in Brazil), Africa (Kenya, Uganda three sites in South Africa), South-East Asia (Bangladesh, three sites in India, two sites in Thailand), and the Western Pacific (three sites in China, Vietnam) from 2018 - 2020. Table S1 provides the number of neonates surveilled over the study period by country. We restricted all analyses to cases within each study with culture-confirmed *K. pneumoniae* sepsis in neonates based on isolates obtained from blood or cerebrospinal fluids (CSF) (Fig. S2).”

E.9) “We quantified the rate of acquisition of antimicrobial resistance genes in K. pneumoniae isolates…” it would be helpful to include details of how you did this

We have expanded our sentence to discuss the methods we used more extensively (lines 39 to 42):

“We analyzed 9,070 *K. pneumoniae* genomes from human isolates gathered globally from 2001 to 2020 to quantify the temporal rate of acquisition of antimicrobial resistance genes in *K. pneumoniae* isolates to predict the future number of drug-resistant cases and deaths that could be averted by vaccination.”

In the spirit of this comment, we have also added more details of this process in the Methods section (lines 231 to 244):

“To estimate the future benefits of such a vaccine, we analyzed the temporal trends in the presence of antimicrobial resistance genes in *K. pneumoniae* by using isolate genomes available on PathogenWatch [31] and in the BARNARDS dataset. We limited our analysis to human isolates from clinical sources that were not taken as rectal or carriage samples (Table S2; Fig. S3). We gathered metadata on the isolates, including their date, source, and country. In total, we analyzed 9,070 *K. pneumoniae* genomes from 68 countries from 2001 to 2020 (Fig. S4-S5). To identify antimicrobial-resistance genes (ARGs) in each genome, we used ABRicate (v1.1.0) with the reference database ResFinder (last updated on June 18^th^, 2022). We fit linear probability model equations to the temporal trends in the number of aminoglycoside-resistant genes (AGRGs) and carbapenem-resistant genes (CRGs) and extrapolated those trends to 2030 (i.e., to predict the future number of AGRGs and CRGs). We used the fit linear probability model to determine the projected increase in ARGs and resistance by 2030 and used that to estimate the increase in the absolute number of aminoglycoside- and carbapenem-resistant neonatal sepsis cases and deaths. ”

E.10) “…predict the future burden…” how do you define “burden”?

We have clarified that by burden we mean number of drug-resistant cases. The new text reads as follows (lines 40 to 43):

“We analyzed 9,070 *K. pneumoniae* genomes from human isolates gathered globally from 2001 to 2020 to quantify the temporal rate of acquisition of antimicrobial resistance genes in *K. pneumoniae* isolates to predict the future number of drug-resistant cases and deaths that could be averted by vaccination.”

E.11) Please quantify the main results with p values, as well as 95% CrIs. When reporting p values please report as p<0.001 and where higher the exact p-value as p=0.002, for example.  If not including p-values, not for the purpose of transparent data reporting please provide reasons as to why not.  
Please ensure that all numbers presented in the abstract are present and identical to numbers presented in the main manuscript text.

In the abstract, we now also include 95%tile CrIs around the key values stated. However, as our objective is to summarize continuous distributions rather than conduct statistical hypothesis testing, CrIs are the preferred and meaningful measure of statistical precision in our study. In the rare cases where we do conduct statistical hypothesis tests (i.e., t-tests to determine the significance of our regression coefficients), we have added p-values (now found in lines 328 - 330 and 355 to 360).

“We expanded our estimates of the effects of a maternal *K. pneumoniae* vaccine on childhood mortality and neonatal sepsis to all countries using regression models as described above (Table S4, *R^2^* of 83.9%, *p* < 0.001).”

“Available *K. pneumoniae* genomes indicate that the prevalence of carbapenemase ARGs (CRGs) is increasing significantly worldwide (Fig. 4A) at a rate of 0.050 [95^th^ percentile linear probability model confidence interval: 0.042, 0.058, *p* < 0.001] ARGs being acquired per isolate yearly (Table S6; *R^2^* = 91.1%). Comparatively, aminoglycoside ARGs (AGRGs) increased significantly in the early 2000s (Fig. 4A), but subsequently have not increased significantly (*p* = 0.436) and are constant at an average of 2.69 AGRGs per isolate.”

E.12) Please provide the actual numbers of events for the outcomes, not just summary statistics/percentages

Did you adjust your analyses for any variable factors? If so, please also detail these

We include actual numbers of events for the outcomes when applicable throughout the text. We did not adjust our analyses for any variable factors.

E.13) In the last sentence of the Abstract Methods and Findings section, please describe the main limitation(s) of the study's methodology.

We have added a sentence addressing this point that reads as follows (lines 51 to 54):

“Nevertheless, our modeling only considers country-level trends in *K. pneumoniae* neonatal sepsis deaths and is unable to consider within-country variability in bacterial prevalence that may impact the projected burden of sepsis.”

AUTHOR SUMMARY
E.14) At this stage, we ask that you include a short, non-technical Author Summary of your research to make findings accessible to a wide audience that includes both scientists and non-scientists. The Author Summary should immediately follow the Abstract in your revised manuscript. This text is subject to editorial change and should be distinct from the scientific abstract. Please see our author guidelines for more information: [https://journals.plos.org/](../../../Downloads/_blank)

Please find here our non-technical Author Summary:

**Author Summary**

Why was this study done?

- Approximately one million newborns yearly die within the first four weeks of life due to bacteria infecting their bloodstream with *Klebsiella pneumoniae* (*K. pneumoniae*) as the leading cause of such infections.
- There have been numerous recent advancements in developing viable *K. pneumoniae* vaccine and antibody-based treatments in animal models, with some treatments reaching Phase 1 clinical trials.
- The impacts of vaccination must be quantified and may prove useful to prioritizing vaccine distribution and better understanding the burden of sepsis.

What did the researchers do and find?

- We present country-specific estimates for the number of deaths and cases and antimicrobial resistance of neonatal sepsis caused by *K. pneumoniae* that would be averted if a vaccine with 70% efficacy were to be added to current vaccines given to pregnant mothers using a Bayesian mixture-model based on clinical surveillance of neonatal sepsis.
- We estimate that a vaccine with 70% efficacy could prevent 399,015 [95^th^ percentile Credible Interval (CrI): 334,523–485,442] cases and 80,258 [CrI: 18,084–189,040] neonatal deaths.
- We find that most cases of *K. pneumoniae* neonatal sepsis are resistant to first-line treatments, such as ampicillin and gentamicin.

What do these findings mean?

- A maternal vaccine that confers newborns with protection from *K. pneumoniae* infection could reduce neonatal sepsis deaths in countries of interest due to all bacteria by nearly 15%.
- This impact would help to achieve targets for improved child health globally and to mitigate inequities in neonatal mortality in low- and middle-income settings.
- Reducing cases of neonatal sepsis may have the additional benefit of reducing antimicrobial resistance by reducing antibiotic use and lowering healthcare utilization and the cost of antibiotics currently used for treatment of newborn sepsis.

METHODS and RESULTS
We ask the following of all authors of modelling studies.  Please see below and in your rebuttal, please sign post to where in the manuscript the relevant information can be located:

E.15) • Please provide a diagram that shows the model structure, including how the disease natural history is represented, the process and determinants of disease acquisition, and how the putative intervention could affect the system.

We have added Figure S6 addressing this comment.

E.16) • Please provide a complete list of model parameters, including clear and precise descriptions of [the meaning of each parameter, together with the values or ranges for each, with justification or the primary source cited, and important caveats about the use of these values noted].

We have included Table S3 with a complete list of model parameters.

E.17) • Please provide a clear statement about how the model was fitted to the data [including goodness-of-fit measure, the numerical algorithm used, which parameter varied, constraints imposed on parameter values, and starting conditions].

When applicable (i.e., fitting regression equations for extrapolations), we present goodness-of-fit statistics and details about the regression scheme used. We add the necessary text to our Methods and Results sections.

In the Methods section when explaining the regression scheme used (lines 227 to 230):

“We related the total number of neonatal deaths due to sepsis (for which estimates are available from CHERG) to the estimated number of avertable neonatal deaths if a *K. pneumoniae* vaccine were distributed as described above using standard ordinary least squares.”

In the Results section (lines 328 to 330).

“We expanded our estimates of the effects of a maternal *K. pneumoniae* vaccine on childhood mortality and neonatal sepsis to all countries using regression models as described above (Table S4, *R^2^* of 83.9%, p < 0.001).”

Lines 354 to 358:

“Finally, we project the benefits of a maternal vaccine in future scenarios of increased AMR prevalence. Available *K. pneumoniae* genomes indicate that the prevalence of carbapenemase ARGs (CRGs) is increasing significantly worldwide (Fig. 4A) at a rate of 0.050 [95^th^ percentile linear probability model confidence interval: 0.042, 0.058, *p* < 0.001] ARGs being acquired per isolate yearly (Table S6; *R^2^* = 91.1%).”

As referenced above, we also add Table S4 and S6 to summarize the regression equations used in the paper.

E.18) • For uncertainty analyses, please state the sources of uncertainties quantified and not quantified [can include parameter, data, and model structure].

We have included text addressing this point in our Methods section (lines 216 to 220):

“To correctly propagate uncertainty across combination products (i.e., when trying to determine the number of cases from the number of deaths using the CFR), we individually multiplied independent samples from the distributions for *p_s,l_* and CFR. We summarized the resulting distribution using the median and 95% credible intervals. We use this approach throughout for propagating uncertainty.”

E.19) • Please provide sensitivity analyses to identify which parameter values are most important in the model. Uncertainty estimates seek to derive a range of credible results on the basis of an exploration of the range of reasonable parameter values. The choice of method should be presented and justified.

While sensitivity analyses may be of value for testing assumptions in settings where direct quantification is impossible (e.g., for unknown parameters), most of the parameters in our analysis are instead derived from data and direct statistical frameworks (i.e., *p_s,l_*). The only parameters that we assign a value through an assumption are vaccine efficacy and vaccine coverage. We add columns to Table S5 for sensitivity analyses of varying the vaccine efficacy/coverage (since the number of cases responds linearly to both variables).

We have added text explaining this and referencing the new table to the Discussion section (lines 398 to 404):

“There are several potential caveats in our study. Our estimates of averted cases and deaths assume that vaccination rates instantly reach their maximum in all countries—that is, we do not model the reduction in cases of *K. pneumoniae* sepsis as vaccine rollout progresses. To address this, we have conducted sensitivity analyses where we vary the vaccine efficacy/coverage (Table S5). Our estimates for the number of avertable deaths respond linearly to vaccine efficacy and coverage. Other studies that estimate the effect of a hypothetical intervention against a communicable disease have used a similar assumption [33,34].”

E.20) • Please discuss the scientific rationale for this choice of model structure and identify points where this choice could influence conclusions drawn. Please also describe the strength of the scientific basis underlying the key model assumptions.

We have added a description of this point in the Methods section and in the Discussion section. In the Method section (lines 185 to 189):

“We used a mixture-modeling approach rather than aggregating the samples into one beta distribution to account for subpopulation variability across sites within the same country from different studies. To efficiently characterize the resulting distribution, we drew 10,000 samples using Latin hypercube sampling (LHS) [27]. We chose LHS over another distribution sampling technique such as Monte Carlo because LHS converges to the true distribution faster.”

Discussion (lines 387 to 391):

“Our methodology represents an advantage over using just clinical data or a single study to draw conclusions and make estimates: we applied a Bayesian framework to aggregate data from various studies and generate increased predictive power. This approach more accurately reflects the underlying uncertainty and allows meaningful inference and projections, even when there are few reported and annotated cases of culture-confirmed neonatal sepsis.”

E.21) We agree with the reviewer (please see below) that additional information regarding the datasets used would be helpful.  Please revise accordingly, as for the abstract.

We have made the requested change in the revised text. Please see above (E.8) for the text addressing this comment. We have added a description of the datasets to the abstract and to the main text, and we respond directly to the reviewer’s request below.

E.22) Please remove role of the funding source from the end of the methods section and include only in the manuscript submission form

We have made the requested change in the revised text.

E.23) Please ensure that results are quantified with p-values as well as 95% CrIs.  When reporting p-values please report as p<0.001 and where higher, the exact p-value as p=0.002, for example.  If not including p-values, not for the purpose of transparent data reporting please provide reasons as to why.  

Please see our discussion above of where we have addressed this comment.

E.24) Please refrain from discussing your results in the results section (see page 11, second paragraph) and report only your findings.  Please move any discussion of your results to the appropriate section of your discussion

We have removed the section to which we believe the editor is referring (an explanation for why drug-resistance rates do not differ between neonates who died from neonatal sepsis and those who survived). We have moved a limited version of this text to the Discussion section (lines 379 to 384):

“Nevertheless, our modeling suggests that the bacteria causing neonatal sepsis in neonates that eventually die do not have higher antimicrobial or multidrug resistance rates. Instead, resistance is high in all isolates and against all drugs, underscoring neonatal fragility and suggesting that other factors such as access to effective drugs and antibiotic dosing regiments or standard of care may be driving differential mortality [7].”

E.25) Please ensure all abbreviations are clearly defined at first use – ARGs and CRGs, for example on page 13 – I couldn’t see a previous definition but my apologies if I missed it

We thank the editor for pointing this out. We have made the requested change in the revised text and ARGs and CRGs are now defined prior to use (once in the Methods section from lines 238 to 241 and secondly in the Results section from lines 356 to 359).

FIGURES
All the figures are very small and therefore not easily interpretable by the reader.  Please see below for specific comments.  Suggest considering placing a title above each image in the figures to help the reader but we leave this to your discretion.

E.26) Please ensure that each figure is affiliated to an appropriate caption which clearly describes its content without the need to refer to the text. Please ensure that any and all abbreviations are defined within the appropriate legend/caption

We thank the reviewer for pointing out ways in which we may improve our figures. We have made all the requested changes in the revision.

We have clearly defined all abbreviations within each figure.

E.27) Please consider avoiding the use of green or red to make figures more accessible to those with colour blindness

We have removed the use of green and red where possible to make the figures more accessible. We have also adopted color-blind accessible palettes.

E.28) Figure 1: we agree with the reviewers that the figure is not very accessible, text is too small and the presentation of different axes is a bit confusing. Please show the axis beginning at zero. If this is not possible, please show a break in the axis.

We have increased the text size and now show the axis beginning at zero.

E.29) In the figure caption please clearly state the meaning of the bars and whiskers and please define GBD and CHERG

We clearly state the meaning of the bars and whiskers and define GBD and CHERG within the caption.

E.30) Figure 2: all the text in the figures in too small for the reader, including the axis labels.  The error bars are very distracting and too large, they should not meet each other. The disparity in bar sizes makes it very difficult to read the data presented here

We have fixed this in the revised figures.

E.31) Figure 3: As above – these are also very small and as such it is difficult to appreciate the different colour densities which we like.  It may also help the reader to title each of the individual maps within the figure.  Please revise accordingly. Please confirm that the appropriate usage rights apply to the use of the maps. Please see our guidelines for map images: https://journals.plos.org/

We have increased figure size and also add titles for each figure.

The maps used are available through pygal under a GNU general public use license.

E.32) Figure 4: as above, please also define abbreviations - AMR, ARGs, CRGs.  The legend is hard to read and could be overlayed on the graph and enlarges, for example.

Thanks for the suggestions on improving the accessibility of the figures. We have overlayed the legend on the graph. We have incorporated all suggestions. Please see the revised figures attached.

SUPPORTING FILES
E.33) Please ensure that all figures/tables are affiliated to an appropriate caption which clearly describes their contents.  Suggest considering applying some universal formatting to the figures throughout the main manuscript and the supporting files.  A number of bar graphs are presented but each are very different, please revise accordingly

E.34) Kp Appendix – please ensure abbreviations are defined throughout

We have made sure all abbreviations are defined throughout.

E.35) Fig S1 – please ensure colours are accessible to those with colour blindness

We have revised the figure to use a colorblind-friendly palette.

E.36) Fig S3 – please define abbreviations

We have made the requested changes in the revised supplemental.

E.37) Fig S5 – is difficult to interpret without a legend for the colours, please include

In the revised figure caption, we have clarified that the colors are simply for contrast and convey no meaning.

38) Fig S13-S16 - please define the meaning of the bars and whiskers and define the abbreviations GBD and CHERG

We have defined all abbreviations and clarified the meaning of the bars and whiskers.

E.39) Fig S17-20 – see earlier re: size of error bars, colours, uniformity, definitions of all abbreviations

We have made the requested changes in the revised supplemental.

E.40) Fig S7 onwards – captions for the maps insufficiently describes what they show.  What is meant by lower and upper bound, for example – you can work it out, but it’s not obvious to the reader.  Do these bounds need to presented as maps? Perhaps a table would be preferable here?

We have clarified what we mean by the lower and upper bound and made the captions more descriptive to properly detail what is shown.

** We note that there are a lot of figures presented here and (as above) we suggest that much of this data would be more accessible if presented as tables **

E.41) What are the items listed as 1-9 above where you refer to a CSV file for the “complete table”.  Please clarify

In order to make our results visually interpretable and intuitive without requiring a reader to open a table and produce their own plot, we have included Figures S8 – S13. Nonetheless, these same values are represented in Table S5 (previously Table S1). We have explained this in our revision with a new caption for Table S5:

“Impact of maternal vaccination by country. Country-specific estimates of overall averted cases, deaths, and result on neonatal mortality as well as antibiotic-resistant deaths. Items 1-9 detail the quantities recorded in the XLSX file for the table.”

DISCUSSION
E.42) Please remove the declaration of interests and data sharing statements form the end of the discussion – these should be included in the relevant parts of the manuscripts only – they will be complied as meta-data

We have made the requested change in the revised text.

REFERENCES
E.43) Please ensure that your referencing follows PLOS Medicine’s style.  Further details can be found here: [https://journals.plos.org/](../../../Downloads/_blank)
In the bibliography please ensure that you list up to more but no more than 6 author names followed by et al where more than 6 authors contribute.  Please ensure that journal name abbreviations are those found in the National Center for Biotechnology Information (NCBI) databases.

We have made the requested change in the revised text.

Comments from the reviewers:

R1.1) Reviewer #1: Overall comment:  In general, this is a useful manuscript that brings into focus an important issue, ie how impactful a vaccine against K pneumoniae might be for preventing neonatal sepsis.  One major issue I have is that the data sources and assumptions and initial values that go into the model are not described well, which gives the impression that the results are generated from a "black box." I suggest adding a "Table 1" that clearly spells out model parameters.  Less importantly, there are some style issue with the manuscript that I suggest cleaning up, in particular the inclusion of methods and discussion points in the results section; including discussion points in the results section is problematic, as it blurs the line between what the model is directly showing and authors' opinions or conclusions.  

We thank the reviewer for recognizing the importance of our manuscript. We appreciate the reviewer’s comments and suggestions and have addressed them all. First, to directly address the reviewer’s request, please see Fig. S6 and Table S3 which describe the model structure and parameters. We address the other points below.

R1.2) Abstract:  Fix the introduction paragraph to clarify that the focus is on neonatal sepsis, not sepsis generally, and that data sources were primarily from LMIC.

We have edited the abstract to address this point (lines 19 to 29):

“Despite significant global progress in reducing neonatal mortality, bacterial sepsis remains a major cause of neonatal deaths. *Klebsiella pneumoniae* (*K. pneumoniae*) is the leading pathogen globally underlying cases of neonatal sepsis and is frequently resistant to antibiotic treatment regimens recommended by the World Health Organization (WHO), including first-line therapy with ampicillin and gentamicin, second-line therapy with amikacin and ceftazidime, and meropenem. Maternal vaccination to prevent neonatal infection could reduce the burden of *K. pneumoniae* neonatal sepsis in Low- and Middle-Income Countries but the potential impact of vaccination remains poorly quantified. We estimated the potential impact of such vaccination on cases and deaths of *K. pneumoniae* neonatal sepsis and project the global effects of routine immunization of pregnant women with the *K. pneumoniae* vaccine as antimicrobial resistance increases.”

R1.3) Page 6, data section:  CHAMPS is Child Health and Mortality Prevention Surveillance (ie, not Childhood…).  Also, the authors should include a sentence or two about each data source so that readers do not need to hunt to find basic information (e.g., countries involved, who was included, method of enrollment, years, etc).

We appreciate the reviewer’s suggestion to improve our discussion of the data used. We have corrected our definition of CHAMPS and added text in both the Abstract and data subsection (also referenced in E.8) addressing this issue. In the Abstract where we discuss all data sources in aggregate and the data used in each phase of the modeling (lines 34 to 43):

“To parameterize our model, we used data from three global studies of neonatal sepsis and/or mortality undertaken in mainly low- and middle-Income countries across all WHO regions (Ethiopia, Kenya, Mali, Mozambique, Nigeria, Rwanda, Sierra Leone, South Africa, Uganda, Brazil, Italy, Greece, Pakistan, Bangladesh, India, Thailand, China, and Vietnam) Across these studies, 26.95% (out of 2330) of fatal neonatal sepsis cases were culture-positive for *K. pneumoniae.* We analyzed 9,070 *K. pneumoniae* genomes from human isolates gathered globally from 2001 to 2020 to quantify the temporal rate of acquisition of antimicrobial resistance genes in *K. pneumoniae* isolates to predict the future number of drug-resistant cases and deaths that could be averted by vaccination.”

In the data subsection of the Methods section (lines 148 to 163):

“CHAMPS, initiated in 2016, conducts autopsies on children under five to determine causal drivers of death at eight study sites in seven low- and middle-income countries (LMICs) across Africa and South-East Asia (Bangladesh, two sites in Ethiopia, Kenya, Mali, Mozambique, Sierra Leone, and South Africa). In contrast, BARNARDS and NeoObs are birth-cohort studies that tracked neonates prospectively through their first month of life, evaluating incidence of sepsis, the antibiotic susceptibility of associated isolates, and resulting mortality. BARNARDS surveilled neonates across 12 study sites in seven countries in Africa and South-East Asia (two sites in Bangladesh, one in India, two in Pakistan, Ethiopia, three in Nigeria, two in Rwanda, and one in South Africa) from 2015 to 2017. Finally, NeoObs reported neonatal sepsis cases, deaths, and antibiotic susceptibility of the isolated bacteria in 19 sites across 11 countries in Europe (Italy, Greece), Latin America (two sites in Brazil), Africa (Kenya, Uganda three sites in South Africa), South-East Asia (Bangladesh, three sites in India, two sites in Thailand), and the Western Pacific (three sites in China, Vietnam) from 2018 - 2020. Table S1 provides the number of neonates surveilled over the study period by country. We restricted all analyses to cases within each study with culture-confirmed *K. pneumoniae* sepsis in neonates based on isolates obtained from blood or cerebrospinal fluids (CSF) (Fig. S2).”

R1.4) Methods, general comment:  One tricky issue with maternal immunization as a method to prevent neonatal infections is that babies born preterm are often more susceptible to sepsis and may need to stay in hospital for medical support until they are large enough to go home, which results in exposures to hospital-associated pathogens, including K pneumoniae.  Conversely, optimal transfer of antibodies from mom to baby across the placenta occurs late in pregnancy (3rd trimester).  Did you account for this issue in the model?  I was curious why you selected 70% efficacy, and whether this figure accounted for less benefit in preterm births or imperfect coverage of serotypes or just assumed that efficacy of the vaccine against sepsis caused by vaccine serotypes would be about 70% in term infants.  I would include this information in the methods section.

We agree with the reviewer’s point that there are challenges in maternal immunization as a strategy to prevent neonatal infections. Nonetheless, it is challenging to precisely quantify how any number of the points the reviewer mentioned may impact vaccine efficacy. The estimate of 70% efficacy is based on a conjugate vaccine candidate that targets the 15 most common K. pneumoniae capsular serotypes that cause invasive infections in neonates. Current sero-epidemiology suggests that these serotypes account for approximately 70% of neonatal sepsis cases. Additionally, the reviewer is correct in pointing out that pre-term infants are at greater risk for neonatal sepsis, and indeed this is what is observed in surveillance data from low and middle-income countries among neonates who experience *K. pneumoniae* sepsis. Optimal transplacental transfer of antibodies occurs later in the third trimester—so efficacy might also be impacted by pre-term births, which is also reflected in the vaccine efficacy of 70%.

We also conduct sensitivity analyses of different vaccine efficacy (50% and 90%; found in Table S5) and produce estimates of averted cases and deaths in either of these scenarios, in case real-world vaccine efficacy ends up varying from what is projected. Finally, we note that vaccine efficacy is not the only factor that determines the number of averted cases and deaths: we also include vaccine coverage. We model effective vaccine coverage as being equivalent to that of the maternal tetanus vaccine, a maternal immunization program that has dealt with some of the challenges of a maternal immunization program. By using that program as a template, we aim to implicitly model some of the issues with a maternal immunization program.

We have added the following text to the Methods section (lines 201 to 205):

“For modeling purposes, we assumed that the efficacy of the vaccine would be 70%: this estimate is based on a conjugate vaccine candidate that targets the 15 most common *K. pneumoniae* capsular serotypes that cause invasive infections in neonates. Current sero-epidemiology suggests that these serotypes account for approximately 70% of neonatal sepsis cases [14].”

R1.5) Results, bottom of page 11.  Here the authors have included text that should be in the discussion section, as it seems to include conclusions drawn from the data.  For example, this is the wording:
" Nevertheless, we do not find overall trends in isolates from neonates who succumbed to sepsis
having higher rates of AMR (Fig. 2A, 2B), resistance to ampicillin or gentamicin (Fig. 2C, 2D)
or higher multi-drug resistance (MDR) (Fig. 2E, 2F), indicating that antibiotic resistance of the
disease-causing K. pneumoniae alone may not significantly impact neonatal mortality. Instead,
resistance is high in all isolates, against all drugs, suggesting that other factors such as access to
effective drugs and antibiotic dosing regiments may be driving differential mortality."

I'm trying to understand how the authors came to this conclusion, as the isolates that were collected from living babies with sepsis may have indeed caused their death (unclear if the study followed them until the end of their illness) or perhaps there was some other issue (e.g. contamination).  Also, since the isolates from deceased children and living children came from two different studies with different patient populations and treatment settings, I would encourage caution in this interpretation.  Also, what antibiotics each child received is likely unknown.

We thank the reviewer for this comment. We have moved the text to the Discussion section and also urge caution in the interpretation of this result. Please note that only BARNARDS and NeoObs conducted antimicrobial susceptibility testing (as discussed in the Methods section from lines 151 to 154), so the isolates all came from living children. Finally, we do acknowledge that we did not know what antibiotics each child received. Please see here the revised text in the Discussion section (lines 379 to 384):

“Nevertheless, our modeling suggests that the bacteria causing neonatal sepsis in neonates that eventually die do not have higher antimicrobial or multidrug resistance rates. Instead, resistance is high in all isolates and against all drugs, underscoring neonatal fragility and suggesting that other factors such as access to effective drugs and antibiotic dosing regiments or standard of care may be driving differential mortality [7]. Regardless, our estimates indicate that maternal vaccination against *K. pneumoniae* would have global benefits and improve efforts towards reaching child survival targets in LMICs worldwide.”

R1.6) Page 12, 5th line under "Projected Global…" heading:  typo with the comma placement in the last number in this line.

We have corrected this typo in the revised text.

R1.7) Page 12, 3rd line from the bottom:  How confident do you feel about extrapolating to North America and Europe from the data sets that included little to no data from high income settings?  Should those settings be excluded, or more data sources added to better support numbers from high income settings?

R1.8) Page 16, discussion:  In the limitations paragraph, the authors mention that the data included did reflect entire countries, but the data sources only included data from a limited number of countries overall.  Isn't the complete lack of data from many countries also an issue that should be mentioned?  For example, were Europe and North America extrapolated from Greece and Brazil, respectively?

We have responded to these two comments jointly as they both target the same underlying point, and after further consideration of the reviewer’s point, we have decided to remove our extrapolation to high-income countries in the main text.

We agree with the reviewer that the extrapolation process is a limitation of our work. In the Discussion section, we write that additional surveillance on the number of neonatal sepsis deaths by country is needed (lines 440 to 443):

“We also lacked data from many countries. We tried to minimize the effect of this by projecting the number of averted cases by country of maternal vaccination on antimicrobial-resistant *K. pneumoniae* neonatal sepsis on a regional basis and limiting our extrapolations to LMICs.”

Regarding the second point made by the reviewer, we agree that there are flaws with any extrapolation procedure. However, the within-sample fit for the regression equation used to do the extrapolation here is decent (*R^2^* > 80%) and indicates that our estimates are least somewhat informative for detailing the benefits of maternal *K. pneumoniae* vaccination on neonatal sepsis. Moreover, while additional data to better understand the drivers of *K. pneumoniae* neonatal sepsis would be ideal, in the absence of that information, we can only conduct simple extrapolations that make assumptions on the prevalence of *K. pneumoniae* neonatal sepsis. We have tried to minimize the impact of over-extrapolating by (a) using a uniform prior in the Bayesian model as discussed above and (b) tailoring our extrapolations to region-level trends in AMR. We have added text in the Discussion section that highlights this limitation (lines 443 to 448):

“Moreover, we used a uniform prior over the probability of mortality from *K. pneumoniae* to consider the variability in *K. pneumoniae* mortality across all countries in a region: this ensures that the credible intervals for our projections to other countries include other mortality rates that may be the true underlying mortality rate for that country. Nonetheless, additional data on *K. pneumoniae* neonatal sepsis across more countries is required to reduce the uncertainty in our estimates.”

R1.9) Figures, general comment: The pictures are quite small relative to the text, so I find myself having to zoom in to interpret the figure then zoom out to read the information about the figure.  Perhaps the journal has a way to fix this mismatch, or maybe the labeling on the figures could be larger.

We have revised all the figures for accessibility. Please see the revised figures for larger text size.

R1.10) Figure 1.  Is there something problematic with the way the model is handling number of deaths for Greece?  The B figure suggests the vaccine is causing deaths, although with the log scale and no zero it's difficult to tell, and the figure with percentages suggests a small percent of deaths are prevented.   

We thank the reviewer for bringing this to our attention. Indeed, this is an artefact of the plotting schema with the log scale and no zero. We have addressed this point in the revised figure.

Although our model projects that there would be the fewest averted deaths in Greece, we do not estimate that there would be none or zero. The low number of averted deaths is driven by the low burden of *K. pneumoniae* neonatal sepsis according to CHERG estimates, but because there are still observed cases and vaccinating would have an impact, our model still estimates that there would be some benefit (0.50 [0.00 – 3.58] deaths averted when using CHERG as the reference data source).

Reviewer #2: SUMMARY
This manuscript reports estimates of the global number of neonatal deaths due to Klebsiella pneumoniae, and the number of these deaths that could be averted by a vaccine with 70% efficacy. In general the approach seems reasonable, though more detail is required on some sections, particularly on how the location-specific estimates were extrapolated to generate regional and global estimates. Specific comments are provided below.

MAJOR ISSUES

R2.1) *    Pages 6-7: the analytic approach appears reasonable if one assumes the fraction of sepsis deaths is homogeneous within the sampled locations, or if the data represent a representative sample of sepsis deaths within each location. It would be useful to examine these assumptions - in particular, where a particular location has several studies, how consistent are these results? If inconsistent, might one explicitly model study-level variation (would lead to greater uncertainty in estimated results, which would be appropriate if there were substantial study-level variation). I think ideally study-level variation would be examined and modelled (if there are enough locations with multiple studies), otherwise this could also be discussed in the limitations.

We thank the reviewer for these helpful comments. To respond to the first part of the reviewer’s comment, we acknowledge that our approach assumes that the data are a representative sample of sepsis deaths within each location. We reference this in our limitations section when we note that our approach may be subject to a sampling bias (lines 432 to 440):

“Moreover, we may be subject to a sampling bias because the data from the three studies we used are point estimates of the impact of sepsis at specific healthcare facilities within a country rather than surveillance over the whole country. In particular, we must limit our analysis to cases of culture-confirmed *K. pneumoniae*, but doing so makes our estimates a probable lower bound on the actual number of averted deaths and cases: it is likely that not all cases of neonatal sepsis are identified as such and cultured. Even if they are cultured, they may not test positive due to low blood volume, a bias whose impact across study sites likely varies appreciably but is unknown [37]. Additional work is required to better understand the factors that impact neonatal sepsis rates within a region.”

To respond to the reviewer’s second request, we consider Fig. S1 (represented in table form below) which shows the raw probability of *K. pneumoniae* infection for neonates dying from neonatal sepsis by study by country. For those locations with multiple studies present, there is general agreement among locations where there are multiple studies present. For instance, in Ethiopia and South Africa, there is close agreement between studies. Nevertheless, we acknowledge that in many other countries, there is greater variability, such as in Bangladesh and more so in India and Kenya where one study registers there being no deaths due to *K. pneumoniae* and another study disagrees. This may also be because different studies have sampled different locations within the country (ex., rural vs urban localities). However, we do not identify a clear trend where one study (i.e., CHAMPS, BARNARDS, or NeoObs) consistently records fewer deaths in countries with multiple study sites (see Table R1 below for evidence). As such, it is challenging to design a model that captures potential study-level bias and variability. We have added a sentence to the limitations section to discuss this (lines 427 to 432):

“We do not know whether heterogeneity within a country is due to external factors or rather indicates study-level variability: given our limited data and few countries for which we have multiple study sites, we do not model study-level variability, though this is an area of future work. Additional work is required to better understand within-country and within-region trends: preliminary data indicates variability in sepsis levels within a country and across private and public hospitals [5,35,36].”

| Fraction of sepsis deaths with *K. pneumoniae* | CHAMPS | BARNARDS | NeoObs |
| --- | --- | --- | --- |
| Bangladesh | 0.42 | 0.1 | 0 |
| Brazil | - | - | 0.25 |
| China | - | - | 0 |
| Ethiopia | 0.64 | 0.69 | - |
| Greece | - | - | 0 |
| India | - | 0 | 0.21 |
| Italy | - | - | 0 |
| Kenya | 0.44 | - | 0 |
| Mali | 0.45 | - | - |
| Mozambique | 0.18 | - | - |
| Nigeria | - | 0.45 | - |
| Pakistan | - | 0.03 | - |
| Rwanda | - | 0.71 | - |
| Sierra Leone | 0.52 | - | - |
| South Africa | 0.25 | 0.27 | 0.11 |
| Thailand | - | - | 0 |
| Uganda | - | - | 0.2 |
| Vietnam | - | - | 0.06 |

Table R1: Fraction of sepsis deaths that were culture-positive for *K. pneumoniae* by location and study. For locations where multiple studies conducted surveillance, the values are colored blue if they are lowest when compared to values recorded by other studies in the same location and red when highest.

R2.2) *    Page 7: for locations with several studies, it is not clear to me why the data were not pooled in a single beta distribution. The approaches will give different answers, though perhaps trivially so, as the current approach adds a value of 1.0 to both beta parameters for each study, via the beta(1,1) prior. I can see the benefit of estimating studies results separately if there was a desire to model study-level variation (I think a beta-binomial model could do this?), yet this is not done (or at least is not described), per comment above.

We appreciate the reviewer’s question and consideration of the details of our statistical methods. To build on our discussion from above, it is exactly because there is no clear trend in one study being biased compared to the others that we do not pool the data into one beta distribution. At a high level, pooling would limit our credible intervals to – in the limit of infinite sampling – zero variance. By using a mixture model, we consider that there may be various mortality rates (one from each study) and that there is not one underlying “true” mortality rate across the entire country (even in the limit of infinite sampling/data). We propagate that uncertainty forward in our subsequent modeling. In other words, if there were a clear alignment in the mortality rate between all studies (after correcting for some degree of study-level variability, as the reviewer suggests), then there should be one identified mortality rate that is consistent among all the studies. The beta distribution would be centered around this mortality rate with a variance reflecting the uncertainty due to limited sampling. However, because we are unable to identify one given mortality rate per country, we use the mixture-modeling approach to capture heterogeneity in the probability of mortality from *K. pneumoniae* represented by each study sampling a different location. We have added a brief justification of this approach to our Methods section (lines 185 to 187):

“We used a mixture-modeling approach rather than aggregating the samples into one beta distribution to account for subpopulation variability across sites within the same country from different studies.”

R2.3) *    Page 8: the regression approach used to extrapolate to other countries is not described in sufficient detail. Given how important this step is to the analysis (going from 18 countries to global), it would be useful to provide additional information on covariates, functional form, evidence of model fit, etc.

We appreciate the reviewer’s suggestion. We have added goodness-of-fit statistics and a table describing each regression equation used in this work (Tables S4 and S6). In the Methods section, we have clarified our discussion of the covariates and functional form (lines 227 to 230):

“We related the total number of neonatal deaths due to sepsis (for which we have estimates in every country from CHERG) to the estimated number of avertable neonatal deaths if a *K. pneumoniae* vaccine were distributed as described above using standard ordinary least squares.”

Finally, we acknowledge that the regression equation used here is imperfect, and we have included a discussion of limitations to our approach (lines 440 to 448):

“We also lacked data from many countries. We tried to minimize the effect of this by projecting the number of averted cases by country of maternal vaccination on antimicrobial-resistant *K. pneumoniae* neonatal sepsis on a regional basis and limiting our extrapolations to LMICs. Moreover, we used a uniform prior over the probability of mortality from *K. pneumoniae* to consider the variability in *K. pneumoniae* mortality across all countries in a region: this ensures that the credible intervals for our projections to other countries include other mortality rates that may be the true underlying mortality rate for that country. Nonetheless, additional data on *K. pneumoniae* neonatal sepsis across more countries is required to reduce the uncertainty in our estimates.”

MINOR ISSUES
R2.4) *    Page 2, abstract: If the word count allows, it would be useful to provide more specific details on the scenarios that produced the vaccine impact estimates (e.g., is this vaccination of all pregnant women globally, or some subset?)

We have added a phrase to the abstract to clarify that the scenario that produced the vaccine impact estimates is with the vaccine being administered with coverage equivalent to the maternal tetanus vaccine (lines 32 to 34):

“We developed a Bayesian mixture-modeling framework to estimate the effects of a hypothetical *K. pneumoniae* maternal vaccine with 70% efficacy administered with coverage equivalent to that of the maternal tetanus vaccine on neonatal sepsis infections and mortality.”

R2.5) *    Page 2, abstract: is it possible to provide some measure of uncertainty around the results presented?

We have addressed this point and added CrIs.

R2.6) *    Page 2, abstract: small point, but it is unclear to me what "The most significant benefits" means. Is this to be read as "largest relative benefit"? Perhaps more specific language would be useful.

We have clarified the text as suggested by the reviewer and directly incorporated this suggestion.

R2.7) *    Page 6: it would be useful to expand the section describing the data, in particular to include the number of samples and distribution across regions.

We have addressed this point and added additional text describing the data. We also have included a supplemental table (Table S1) that provides the sample size of neonates with neonatal sepsis and *K. pneumoniae* by country and by study.

R2.8) *    Page 6 (data): please provide some comment on possible bias that comes with restricting analyses to culture-confirmed cases (and what fraction of cases this generally represents). I realize that using culture-confirmed cases may be the only feasible approach, but still useful to know if there are any biases could result, and how they are dealt with.

While culture is the best available approach for diagnosis of neonatal sepsis, imperfect sensitivity is associated with low blood volume draws, which are more frequent for the youngest infants (see below citation; also referenced in the revised manuscript text). We have described this problem in the revised text and highlighted that, in the event of limited sensitivity, there are likely more cases of neonatal sepsis that could be averted than our model estimates. However, it is challenging to quantify the significance of this bias without an additional study that determines the fraction of sepsis cases with samples obtained, and the impact of blood volume on culture positivity under the real-world conditions in place in each study setting (which may differ appreciably). We have made a note of this in the Discussion section (lines 434 to 439):

“In particular, we must limit our analysis to cases of culture-confirmed *K. pneumoniae*, but doing so makes our estimates a probable lower bound on the actual number of averted deaths and cases: it is likely that not all cases of neonatal sepsis are identified as such and cultured. Even if they are cultured, they may not test positive due to low blood volume, a bias whose impact across study sites likely varies appreciably but is unknown [37].^”^

R2.8) *    Page 7: around "effective coverage level equal to that of the maternal tetanus vaccine", it would be useful to give the median and IQR (or something similar) to give the reader some idea of the average level and variation in vaccine coverage assumed for the analysis.

We appreciate this suggestion and have made the requested revision in the text (lines 199 to 201):

“We determined the number of vaccine-avertable deaths associated with the deployment of a maternal *K. pneumoniae* vaccine at an effective coverage level equal to that of the maternal tetanus vaccine (median: 90%; range 38.5% to 100%) [30].”

R2.9) *    Page 7: I might describe what you used the LHS sample for, for readers who are unfamiliar.

We have added a section in the text describing how the LHS samples are used (lines 216 to 220):

“To correctly propagate uncertainty across combination products (i.e., when trying to determine the number of cases from the number of deaths using the CFR), we individually multiplied independent samples from the distributions for *p_s,l_* and the CFR. We summarized the resulting distribution using the median and 95% credible intervals. We use this approach throughout for propagating uncertainty.”

R2.10) *    Page 10: "The average rate of resistance to gentamicin, which is usually prescribed in combination with ampicillin as the first- line treatment for sepsis per current WHO guidelines,32 is 57.22% [CrI: 31.540-80.42]" - I am a bit surprised by how broad the intervals are. Is this because of a small number of observations (i.e. individuals), or the extrapolation process, or something else?

The reviewer is correct: the large uncertainty intervals are because we have very different rates of gentamicin resistance reported across regions and by study. Since we are reporting statistics averaged over all regions, this creates a large uncertainty interval. The large uncertainty intervals persist even with a large sample size: in this case, over 100 isolates were tested. It is more informative to consider the resistance rates by geography, as we do in the next section: the purpose of this value is to introduce that analysis and provide a simple summary statistic for the antimicrobial resistance of a drug for which there is significant interest in its resistance.

R2.11) On a similar subject, there is substantial uncertainty around Panel A in Figure 1, but not Panels B and C. I realize that B and C are log-scale, but it appears Panel A still has more relative uncertainty (take Thailand for example where in Panel A the interval ranges from ~0 to >3 times the point estimate). Is everything here correct?  

We regret that we do not observe the same issue as to which the reviewer is referring. In particular, for Thailand the value for Panel A is 0.466 [0.008 – 3.867] and 50.32 [0.84 – 417.29] for Panel B. There should be additional uncertainty in Panels B and C because we have incorporated uncertainty from using CHERG/GBD estimates of total neonatal deaths and the CFRs from BARNARDS. Indeed, this is the case.

We acknowledge that plotting the data on a log scale is not ideal as it makes interpretability challenging, but unfortunately the quantities we need to plot span so many orders of magnitude that a log scale is critical to being able to show all the values. Nonetheless, we have tried to address this problem in the revised figures by including a value of 0.

Reviewer #3: According to the authors, there has been no study projecting the effects of K. pneumoniae vaccine distribution. Given that there are numerous efforts in developing a viable K. pneumoniae vaccine, the authors tried to estimate the global, regional, and national impact of a hypothetical vaccine with 70% efficacy on neonatal sepsis infections and mortality using a Bayesian mixture-modeling framework. The estimated impact was presented in terms of number of neonatal sepsis deaths and neonatal sepsis cases averted due to the hypothetical vaccine. In addition, the authors also examined the AMR of sepsis-causing isolates and tried to project the future benefits of maternal K. pneumoniae vaccination. Overall, this is a well-designed and conducted study and the results of the study are of relevance, particularly for LMICs in Africa. Below are my specific comments.

R3.1) Please add line numbers for easier reference.

We have made the requested change in the revised text.

R3.2) The sponsor of the study provided input in the writing of the report, which might result in conflict of interest.

Please see above (E.1) for our response to this comment and addressing this issue.

R3.3) Data used in the study are not fully available.

We have revised the data availability statement. We paste the corrected version that describes the availability of the study data here again for reference (also referenced in E.2):

“The CHAMPS data are freely available online upon request. In this analysis, we used CHAMPS Level 2: De-Identified Data, which are all available at the following link after signing a data transfer agreement: <https://champshealth.org/data/>.

BARNARDS data are owned by the Ineos Oxford Institute for Antimicrobial Resistance. They are freely available upon request from the principal investigators of the study. For details, please reach out to Kathryn Thomson at [kathryn.thomson@zoo.ox.ac.uk](mailto:kathryn.thomson@zoo.ox.ac.uk). NeoObs data are owned by the Global Antibiotic Research and Development Partnership and are freely available upon request. For details, please contact Sally Ellis at [sellis@gardp.org](mailto:sellis@gardp.org).”

R3.4) Page 7, method: "We extended this analysis to estimate the absolute number.. we report values derived using CHERG data." It seems that sometimes you used CHERG data and sometimes you used GBD data to derives the parameter values. However, are data from CHERG and from GBD comparable to each other? Did you do any adjustment to the GBD data to make them comparable to the CHERG data? I did not see you mention this in the method section.

We apologize that we may have been unclear in our text that we only report values using CHERG data after Figure 1. We have made this clear in the revision. We always do the analysis using both CHERG and GBD as reference data sources. In Figure 1 we present the estimates from using both datasets. In all subsequent analyses, we only use CHERG data.

GBD and CHERG represent alternative approaches to measuring the same quantity – the number of deaths by country for various causes (and stratified by age, sex, etc.), although drawing on differing source data. While one option may be to rescale values from GBD to match those from CHERG, we feel it is important to in fact account for variability across the two studies, as it is not known if one source is more reliable than another. Thus, we feel that using data from both CHERG and GBD is our best method of estimating that. Both datasets already estimate the same quantities, and conducting any adjustment of that data would potentially nullify the unique information gathered by getting different estimates when using the two datasets.

R3.5) Page 7, method: "We attempted to minimize the impact of potential.. to estimate the CFR". I don't understand how Bayesian method can help minimize the bias here. Loss to follow-ups are usually informative. I am not sure whether Bayesian method can help reduce the bias due to informative LTFUs.

We thank the reviewer for their comment and pointing out that our text may have been unclear. A Bayesian approach on its own does not minimize bias, and indeed the reviewer is correct that LTFUs are informative. However, by using a uniform prior in our Bayesian approach, we attempt to minimize the impact of bias. In other words, if the actual CFR is different from the measured value due to LTFUs, the use of a uniform prior helps to remove the bias by increasing the width of the uncertainty intervals. This works in both directions: regardless of whether the CFR is overestimated or underestimated, a beta distribution centered around the given mortality rate will always have less probability density in the tails than a uniform distribution, so the use of a uniform prior helps to minimize the impact of bias in our calculated CFRs. We have revised our text to make this more clear (lines 211 to 215):

“Concretely, a uniform prior reduces the impact of a potential bias in the CFR by increasing the width of the credible intervals if the distribution were to instead only be defined by the observed data because a uniform distribution will always have additional and equal probability density in the tails compared to an informative distribution parameterized by data.”

R3.6) Method: To estimate the global, regional, and national number of cases and deaths, why did you not use some hierarchical modeling approach? Bayesian framework should be perfect to incorporate hierarchical data structure, which can make the estimates between different levels internally consistent. However, I did not see the hierarchical structure in the method section.

We appreciate this suggestion from the reviewer. While a Bayesian hierarchical model could also be a way of approaching the analysis to conduct the extrapolation to estimate the global, regional, and national number of cases and deaths from the countries for which we currently have data, doing so would require a knowledge of the data generation process that explains what differentiates (either deterministically or through a stochastic censoring process with a given distribution) those countries for which we have data compared to those for which we do not have data. In other words, use of a hierarchical model requires understanding the data generation process that led to the observed data so that the underlying and complete data can be reconstructed or reverse engineered. Likewise, we should also understand how the specific sampling sites within a country capture the overall burden of *K. pneumoniae*, or the probability distribution of *K. pneumoniae* infection within a country. Unfortunately, all of these factors are unknown. Although we could make some assumptions about the parameters and distributions that would be required for this approach, the need for extensive assumptions would severely undercut the validity of estimates and their value for policymaking. As discussed in the limitations section, there is minimal data about neonatal sepsis and the etiologies causing mortality from neonatal sepsis. There is even further limited data that attempts to address how neonatal sepsis mortality rates may vary within a country and the drivers of differential neonatal mortality. Although we appreciate the reviewer’s suggestion, given the data constraints, at this time implementing a hierarchical model is not feasible.
