# Supplementary material for: Global, regional, and national estimates of the impact of a maternal Klebsiella pneumoniae vaccine: A Bayesian modeling analysis
Source: PLoS Med. 2023 May 22;20(5):e1004239. doi: 10.1371/journal.pmed.1004239 (PMC10270628; doi:10.1371/journal.pmed.1004239)
Supplement: Attachment — Submitted filename: ResponseToReviewers-Final-03202023.docx [file pmed.1004239.s006.docx]

March 20^th^, 2023

PLOS Medicine

Dear Dr. Dobbs,

We thank you and the reviewers for the comments on our manuscript, *Global, regional, and national estimates of the impact of a maternal Klebsiella pneumoniae vaccine: A Bayesian modeling analysis*. We have addressed all points and largely incorporated all suggestions and edits.

We thank the editorial team and reviewers again for their assessment of the manuscript and thoughtful suggestions.

Sincerely,

Ramanan Laxminarayan

Dr. Ramanan Laxminarayan and co-authors

ABSTRACT

Line 17 – please replace the sub-heading “summary” with “Abstract”

We have edited the text as requested.

AUTHOR SUMMARY

Thank you for including an author summary some suggestions are detailed below

Line 69: suggest “pre-clinical model” perhaps instead of “animal”

We have edited the text as requested.

Line 74: To improve accessibility to the reader, suggest the following - “Using a Bayesian mixture-model based on clinical surveillance of neonatal sepsis, we present country-specific estimates for the number of deaths and cases of antimicrobial resistant neonatal sepsis, caused by K. pneumoniae, that would be averted if a vaccine with 70% efficacy was to be given to pregnant mothers.”

We have revised the text with this suggestion.

Line 82: suggest moving this point to precede that at line 79

Thanks for this suggestion. We have made the suggested edit.

Line 85: suggest rewording this statement for improved clarity – “…countries of interest…” is rather vague, perhaps “especially in LMICs” instead, perhaps? And, “…due to all bacteria…” this is a little confusing/vague – do you mean bacteria other than K pneumoniae or all strains of K pneumoniae?

We have edited the text and it now reads as follows (lines 88 – 91):

“A maternal vaccine that confers newborns with protection from *K. pneumoniae* infection could reduce neonatal sepsis deaths in many low- and middle-income countries (LMICs) by nearly 15%.”

Line 88: Suggest “This would help to achieve targets set by […] for improved global child health and to mitigate against inequity in neonatal survival in low- and middle-income settings compared to high-income settings. Suggest that the blank space in brackets is completed (WHO targets?)

Thanks for this suggestion. We have made the suggested edit and use “targets set by the World Health Organization” as the benchmark.

Line 90: Suggest “Reducing cases of neonatal sepsis by vaccination could also contribute to reduced antibiotic use, subsequent improvements in antimicrobial resistance rates and a reduction in healthcare utilization and expenditure.” Or something similar

Thanks for this suggestion. We have made the suggested edit.

METHODS and RESULTS

Please see statistical reviewer (reviewer #3) comments below

We address this comment below.

COMPETING INTERESTS STATEMENT

Line 492: Thank you for updating your statement. Please remove from the end of the discussion in the main manuscript and include only in the manuscript submission form when you re-submit your manuscript.

We have removed the statement from the main text.

FIGURES

Figure 4: in the caption you refer to ARGs (and define them) as well as CRGs (and define these) but CRGs are not detailed anywhere in the legend of figure 4 that is available to me. Please revise as necessary.

Thanks for pointing this discrepancy out. We have revised the caption to read as follows:

“Projected Benefits of a Maternal Vaccine Under Future Antimicrobial Resistance (AMR) Scenarios. A) Average number of antimicrobial resistance genes (ARGs) that confer resistance to aminoglycosides (N = 24,359) and carbapenems (N = 5,189) per isolate by year of collection of K. pneumoniae isolates. B) Projected median number of meropenem-resistant neonatal deaths averted by 2030 due to maternal vaccination based on the trends shown in Fig. 4A. The maps are reprinted from pygal_maps_world under GNU GPL.”

Comments from Reviewers:

Reviewer #1: The authors appear to have addressed my concerns.  I would ask for one simple correction -- CHAMPS does not do autopsies but rather Minimally Invasive Tissue Sampling (MITS).

We have made the suggested edit (line 151 – 155):

“CHAMPS, initiated in 2016, conducts Minimally Invasive Tissue Sampling (MITS) on deceased children under five to determine causal drivers of death at eight study sites in seven low- and middle-income countries (LMICs) across Africa and South-East Asia (Bangladesh, two sites in Ethiopia, Kenya, Mali, Mozambique, Sierra Leone, and South Africa).”

Reviewer #2: Thank you, these revisions resolve my concerns.

Reviewer #3: I appreciate the authors' responses to my comments. I am happy with most of the responses but I cannot agree with their response to my comment #5. I don't think using a uniform prior can help reduce bias due to lost-to-follow up or any bias.. A uniform prior just means that the analyst knows little/nothing about the quantity of interest and rely almost entirely on the data to inform the estimate. It dose not help with reducing bias at all, and increasing the uncertainty of the estimate does not mean reducing bias either. It just means that you are more uncertain about the estimate.

We thank the reviewer for this point. We have removed the discussion of the uniform prior reducing the impact of bias in the methods and discussion sections. Our new text regarding the use of the uniform prior when estimating the CFR reads as follows (lines 215 – 219):

“We used the same Bayesian beta framework with a uniform prior described above (we treated whether the neonate died from *K. pneumoniae* or recovered as the binomial event for which we seek to estimate the probability parameter) to estimate the CFR.”

We also add a sentence in the discussion section from lines 418 – 420 that address LTFU as a caveat of our work:

“Finally, our estimates of the CFR of *K. pneumoniae* neonatal sepsis may be biased due to loss-to-follow-up: in particular, we are unable to account for whether there is a sampling bias of a disproportionate number of deaths being recorded. Further data is required to address this issue.”
